# Deciphering Electrocatalytic Activity in Cu Nanoclusters: Interplay Between Structural Confinement and Ligands Environment

**DOI:** 10.1002/smll.202500302

**Published:** 2025-03-06

**Authors:** Sourav Biswas, Yamato Shingyouchi, Maho Kamiyama, Milan Kumar Jena, Masaki Ogami, Tokuhisa Kawawaki, Biswarup Pathak, Yuichi Negishi

**Affiliations:** ^1^ Research Institute for Science & Technology Tokyo University of Science Tokyo 162‐8601 Japan; ^2^ Department of Applied Chemistry Tokyo University of Science 1‐3 Kagurazaka, Shinjuku‐ku Tokyo 162‐8601 Japan; ^3^ Department of Chemistry Indian Institute of Technology Indore Indore Madhya Pradesh 453552 India; ^4^ Institute of Multidisciplinary Research for Advanced Materials Tohoku University Katahira 2‐1‐1, Aoba‐ku Sendai 980‐8577 Japan

**Keywords:** atomically‐precise, copper, copper nanoclusters, CO_2_ reduction, nanoclusters

## Abstract

Ligand‐protected copper nanoclusters (Cu NCs) with atomic precision have emerged rapidly due to their fascinating structural architectures and versatile catalytic properties, making them ideal for investigating structure–activity relationships. Despite their potential, challenges such as stability issues and limited structural diversity have restricted deeper exploration. In this study, three distinct Cu NCs are synthesized using a one‐pot reduction strategy by carefully modifying reaction conditions. Intriguingly, the same *p*‐toluenethiol ligand produces two different geometries, while varying ligands with *m*‐aminobenzethiol—yielded clusters with similar geometric architectures. These NCs are evaluated for electrocatalytic CO_2_ reduction, uncovering diverse catalytic activities and product selectivity. Experimental and theoretical analyses reveal that the interplay between the core structure confinement and surface ligand environment governs their catalytic behavior. Specifically, the Cu_11_ NC with *p*‐toluenethiol ligand exhibits selectivity toward HCOOH production (FE_HCOOH_∼45% at −1.2 V vs RHE), whereas substituting *p*‐toluenethiol with *m*‐aminobenzethiol shifted the selectivity to the competitive side reaction (FE_H2_∼82% at −1.2 V vs RHE). Conversely, altering the geometry of Cu_18_ NC while retaining the *p*‐toluenethiol ligand decreases such selectivity (FE_HCOOH_∼35% at −1.2 V vs RHE). These findings highlight the tunability of Cu NCs for tailored catalytic applications through precise control of their structure and surface chemistry.

## Graphical abstract

Three distinct Cu nanoclusters are synthesized and investigated for their electrocatalytic performance in CO₂ reduction. Among these, two clusters with similar ligands exhibit different geometries, while another pair, despite possessing diverse ligands, share a comparable structural framework. This interplay of structural and compositional variations provides critical insights into the structure‐property relationship, ultimately influencing the product selectivity in the CO₂ reduction reaction.

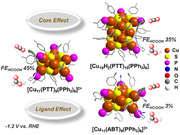

## 1 Introduction

Electrochemical reduction of carbon dioxide (CO_2_) represents a promising approach to advancing carbon neutrality in a sustainable manner.^[^ ^1^ ^]^ However, scaling up electrochemical CO_2_ reduction reaction (CO_2_RR) for practical applications hinges on the development of efficient catalysts.^[^ ^2^ ^]^ In this area, transition metal‐based catalysts have garnered significant interest due to their tuneable electrochemical properties, which align with the reduction potentials required for converting CO_2_ into desirable products.^[^ ^3^ ^]^ Initially, transition metal complexes gained significant attention due to their tuneable electronic structures.^[^ ^4^ ^]^ However, recent advancements have shown that transition metal nanoparticles exhibit superior catalytic activity, primarily due to their unique intrinsic surface properties.^[^ ^5^ ^]^ Interestingly, further research has revealed that the structural architecture of these catalysts plays a crucial role in determining their efficacy however controlling such architectures in transition metal‐based nanoparticles is challenging. This realization has paved the way for the utilization of transition metal‐based nanoclusters (NCs) with precisely defined structural features and optimized surface properties for this purpose.^[^ ^6^ ^]^

Initially, noble metal‐based NCs and their alloys were widely used for this application.^[^ ^7^ ^]^ However, despite their well‐defined architectures and notable surface properties, these NCs predominantly produce CO as the main CO₂RR product, limiting their versatility.^[^ ^7^, ^8^ ^]^ In contrast, copper (Cu) NCs stand out due to their unique ability to catalyze CO_2_RR into a wide array of products, including hydrocarbons and oxygenates due to their ability to stabilize the intermediates.^[^ ^9^ ^]^ This distinctive behavior makes Cu NCs a key focus in the field.

Despite their promise, studies on Cu NCs for CO₂RR remain limited. The pioneering work in this field, initiated by Tang et al., demonstrated that Cu NCs exhibit formic acid (HCOOH) selectively at lower overpotentials.^[^ ^10^ ^]^ However, as the applied potential increases, the selectivity shifts toward the hydrogen evolution reaction (HER). They further identified that the presence of lattice hydrides in Cu NCs plays a crucial role in promoting product selectivity. However, Li et al. identified another Cu NC containing the highest metal‐to‐hydride ratio that predominately produces HCOOH.^[^ ^11^ ^]^ On the other side, Liu et al. synthesized three distinct hydrides free Cu_8_ NCs by systematically varying the surface‐protecting ligands.^[^ ^12^ ^]^ These variations in ligands altered the arrangement of the metal ions, resulting in two distinct geometric structures. They discovered that twisted‐cubic Cu_8_ NCs favored the HER, whereas ditetrahedron‐shaped Cu_8_ NCs predominantly produced HCOOH. Thus, the above findings cumulatively identified the effect of various factors but there is no clear understanding of whether the presence of hydrides, the change in the structural architecture, and the alteration in surface ligand have any individual effect on the selectivity of the products. Expanding the understanding of ligand effects, Wu et al. revealed that the precise design of ligand structures enables Cu NCs to exhibit selective production of various hydrocarbons.^[^ ^13^ ^]^ Our previous research was focused on the role of structural defects in Cu NCs that significantly influence their selectivity in CO₂RR products, providing an additional parameter for optimizing catalytic performance.^[^ ^14^ ^]^ So, the utilization of new Cu NCs in electrochemical CO₂RR is rapidly expanding with diverse structural architectures and related properties. However, the ultimate goal of creating industrial‐grade catalysts remains unmet.

This highlights a significant knowledge gap concerning the precise structural features or functional groups necessary for the product selectivity. Addressing this requires comparing the catalytic efficacy of NCs with similar geometries but different ligands, as well as those with consistent ligands but distinct geometries. However, independently controlling NCs geometry while maintaining uniform ligands, or modifying ligands without altering the geometry, remains challenging.^[^ ^15^ ^]^ Ligands coordinate metal atoms, often impacting the structural framework, making it difficult to isolate these variables. As a result, generating NCs with either uniform geometries and varied ligands or consistent ligands and diverse geometries is particularly complex, limiting precise comparisons of their catalytic performance.

However, in this study, we strategically synthesized three distinct Cu NCs using a meticulously designed one‐pot reduction method. The synthesized NCs include [Cu_11_(PTT)_9_(PPh_3_)_6_]^2+^ (Cu_11_PTT) (PTT: *p*‐toluenethiolate) and [Cu_11_(ABT)_9_(PPh_3_)_6_]^2+^ (Cu_11_ABT) (ABT: *m*‐aminobenzethiolate) NCs, which share a similar structural architecture but differ in their thiolate ligand systems, and [Cu_18_H_2_(PTT)_15_(PPh_3_)_6_] (Cu_18_PTT) NC, which features the same PTT ligand as Cu_11_PTT but adopts a distinct structural architecture. These variations were achieved by precisely controlling the reduction conditions during the synthesis process. Although both Cu₁₁ NCs share the same geometry, differences in their ligands significantly impact cuprophilic interactions within the clusters, directly affecting the core confinement. In contrast, Cu_18_PTT demonstrates how even with the same PTT ligand, adjustments in the reduction conditions can lead to a rearrangement of the core's structural architecture. This rearrangement is also driven by modified cuprophilic interactions, emphasizing the critical role these interactions play in defining the core geometry. The alteration in cuprophilic interactions has profound implications for the catalytic properties of these NCs. Changes in core length and structural arrangement directly affect the active catalytic sites, thereby influencing the efficacy and selectivity of the catalytic processes. Furthermore, the nature of the surface ligands plays a pivotal role in dictating product selectivity, highlighting the importance of ligand choice in tailoring catalytic performance. Thus, this study not only demonstrates a precise strategy for designing Cu NCs with tailored structural architectures by controlling their reduction conditions but also elucidates the significant impact of structural variations and surface ligand modifications on catalytic behavior. The findings also underscore how subtle changes in synthesis parameters and ligand systems can lead to optimized catalytic applications with enhanced product selectivity.

## 2 Results and Discussion

### 2.1 Investigating One‐Pot Reduction Strategies for Diverse Products

**Scheme 1:**
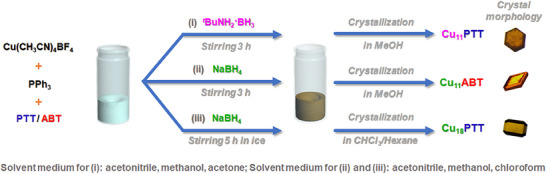
Schematic representation of the one‐pot reduction approach for the synthesis of these three different NCs which resulted in different crystal morphology visible in the optical microscopic image.

All three Cu NCs were synthesized using a one‐pot direct reduction strategy, as depicted in **Scheme** **1**. Generally, in NCs synthesis, the choice of ligand plays a pivotal role in directing the assembly of metal ions, as the interaction between metal ions and ligands determines the nuclearity and structural architecture of the resulting NCs.^[^ ^15^, ^16^ ^]^ However, in this study, the carefully optimized synthetic protocols consistently yielded NCs with the same number of Cu(I) atoms in their assemblies, regardless of the variation in ligand systems. Interestingly, the study also demonstrates that altering the reaction conditions while keeping the ligand system constant can result in NCs with distinct structural geometries. In a typical synthetic route, hexagonal yellow crystals of Cu_11_PTT NC were obtained by reacting a Cu(I) precursor with PTT and PPh_3_ ligands in a CH_3_CN medium, using *^t^* BuNH_2_·BH_3_ as a mild reducing agent. During the transition from PTT to ABT as the ligand system, maintaining the same number of Cu(I) atoms in the resulting NC required strategic modifications to the reaction conditions. This was achieved by replacing the mild reducing agent with a stronger one and adjusting the solvent medium accordingly. Specifically, the use of NaBH_4_ significantly altered the reaction dynamics, leading to the formation of yellow‐colored rhombohedral crystals of Cu_11_ABT NC while preserving the nuclearity of the NC. Conversely, when the reducing agent was altered at the initial reaction by retaining the same PTT ligand, a notable rearrangement occurred in the number of Cu atoms forming the NC. This change in reaction dynamics resulted in the synthesis of Cu_18_PTT, a yellow‐colored NC with an elongated square gyrobicupola‐like crystal morphology. These observations highlight the intricate interplay between the reducing agent and ligand in dictating the nuclearity and geometry of the resulting Cu NCs.

### 2.2 Unveiling Geometric Insights Through Structural Analysis

**Figure 1:**
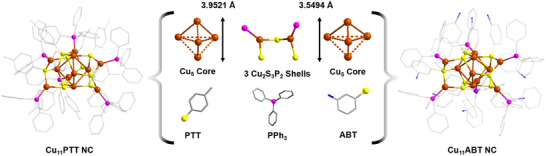
Structural anatomy of Cu_11_PTT and Cu_11_ABT NCs where both exhibit core‐shell morpholgy but a difference in their core size is persistent. Hydrogen atoms are removed from the ligands for clarity. Color legend: Cu, brown; S, yellow; P, violet; N, blue; and C, gray stick.

All Single‐crystal X‐ray diffraction (SCXRD) analysis offered comprehensive structural insights into these three NCs (Tables S1–S3, Supporting Information). The analysis revealed that Cu_11_PTT crystallizes in a hexagonal crystal system, specifically adopting the space group *P6_3_* (No. 173). In contrast, both Cu_11_ABT and Cu_18_PTT NCs display distinct crystallographic characteristics, crystallizing in a triclinic crystal system with the space group *P‐1* (No. 2). Detailed structural analysis of the Cu_11_PTT NC unveils an intriguing arrangement where a Cu_5_ triangular bipyramid core is intricately enveloped by three Cu_2_S_3_P_2_ motif units (**Figure** **1**). Within the core, the average Cu─Cu distance measures 3.0082 ± 0.0745 Å, indicative of a weak coprophilic interaction where the total length of the core is 3.9521 Å.^[^ ^17^ ^]^ In the surrounding motif shell, the Cu─S bond lengths ranging from 2.2431 to 2.3092 Å (average 2.2818 ± 0.0133 Å), while the Cu─P bond lengths vary between 2.1976 and 2.2268 Å (Average 2.2104 ± 0.0044 Å). It has been observed that each thiolate ligands coordinate with three Cu atoms, yet there is a discernible discrepancy in their choice of coordinated Cu atoms between core and shell. As, each motif shell contains two terminal thiolate ligands, which are additionally anchored to two Cu atoms from the core. Conversely, the middle thiolate ligand, bridging the two terminal Cu atoms of the motif, interacts with only one Cu atom from the core. This intricate coordination pattern underscores the complexity and precision of the Cu_11_PTT NC architecture. Although the alteration of PTT ligands by ABT does not change the overall core‐shell structural geometry of the NC, but we identified a relatively confined core structural architecture (Figure 1). The average Cu─Cu distance measures 2.8384 ± 0.0132 Å within the Cu_5_ core, indicates relatively a stronger coprophilic interaction which ultimately reduces the total length of the core to 3.5494 Å than previous. In the surrounding motif shell, the Cu‐S bond lengths range from 2.2224 to 2.2936 Å (average 2.2566 ± 0.0073 Å), while the Cu─P bond lengths vary between 2.2009 and 2.2178 Å (Average 2.2093 ± 0.0022 Å).

**Figure 2:**
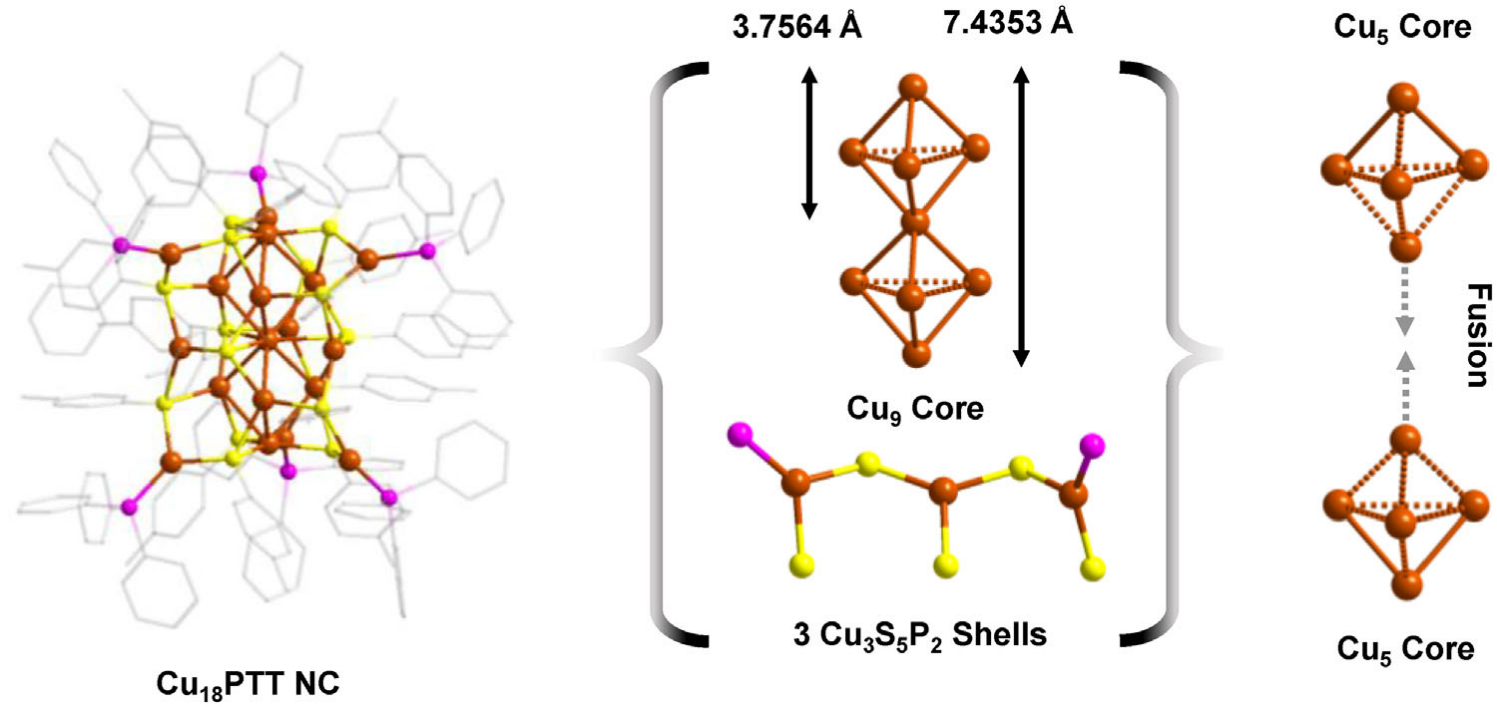
Structural anatomy of Cu_18_PTT NC which exhibits a core‐shell morpholgy where the core and shells are formed by the fusion process. Hydrogen atoms are removed from the ligands for clarity. Color legend: Cu, brown; S, yellow; P, violet; and C, gray stick.

However, in the case of Cu_18_PTT NC, although it exhibits a core‐shell architecture but an alteration of its core and shell is quite distinct than previous (**Figure** **2**). It exhibits a Cu_9_ core which is intricately surrounded by three Cu_3_S_5_P_2_ motif units. Interestingly, the details structural analysis revealed that the Cu_9_ core is formed by fusion of two Cu_5_ triangular bipyramidal geometries through a vertex Cu atom sharing (Figure 2). So, the change in the reducing agent expand the NC architecture by dimerization of the core. Within the Cu_9_ core, the average Cu‐Cu distance is ≈2.6825 ± 0.011 Å indicates relatively stronger cuprophilic interactions compared to Cu_11_PTT NC's core, suggesting a more compact and robust structure. The total length of this fused core extends to ≈7.4353 Å, whereas the length of one Cu_5_ unit is 3.7564 Å. This suggests that the kernel fusion not only enlarges the core but also confines it through stronger cuprophilic interactions. Although the fusion confines the core geometry but the change of the PTT ligand with ABT, the confinement effect is more prominent which squeezed the core with much effectively. So, the confinement of the core Cu atoms mostly depends on the electronic environment of the surface protecting ligands and the overall geometrical architecture of the NC. Additionally, the fusion process extends to the motif units of Cu_18_PTT NC. Here, two Cu_2_S_3_P_2_ motif units fused via a terminal Cu atom to create a modified Cu_3_S_5_P_2_ motif unit. This modification results in the loss of one triphenylphosphine unit, a change facilitated by the inward orientation of the shared Cu atom, which discourages further attachment of the bulky ligand. Thus, as the core size increases due to kernel fusion, there is a corresponding evolution in the motif units to appropriately complement the enlarged core. Regarding bond lengths, the Cu─S bonds within these motif units range from 2.2114 to 2.3722 Å (average 2.2644 ± 0.0072 Å). The Cu─P bond lengths show less variation, ranging from 2.1943 to 2.2119 Å (average 2.2023 ± 0.0023 Å). Interestingly, these measurements indicate that the bond lengths between the Cu_11_ and Cu_18_ NCs do not undergo significant changes despite the structural modifications within the core and motifs. Finally, the overall cluster size of the Cu_18_ NC does not increase substantially, which can be attributed to the motif units adopting a zigzag pattern. This configuration effectively accommodates the expanded Cu_9_ core, thus optimizing the spatial arrangement of the cluster. This detailed arrangement demonstrates the nuanced interplay between core expansion and motif adaptation, which together contribute to the stability and functionality of the Cu_18_PTT NC.

Upon a detailed comparison with the existing literature, a remarkable resemblance is observed between the structural architecture of the Cu_11_ NCs and the previously reported [Cu_11_(TBBT)_9_(PPh_3_)_6_]^2+^ (Cu_11_TBBT) (TBBT: 4‐tert‐butylbenzenethiolate) NC which was synthesized by using NaBH_4_ as reducing agent.^[^ ^18^ ^]^ A comparison of the synthetic routes highlights the influence of ligand electronic and steric effects. The PTT ligand, leveraging its hyperconjugation and inductive effects, enhances electron donation to Cu(I) atoms, enabling the formation of the initial complexation and allowing NC synthesis with a mild reducing agent. In contrast, the ABT and TBBT ligands, where the initial complexation is primarily governed by inductive effects, require stronger reducing agents for NCs formation. Although, all Cu_11_ NCs exhibit similar core geometries and motif arrangements, a weaker cuprophilic interaction is evident in the core geometry of Cu_11_TBBT NC, likely due to differences in the thiolate ligands employed, resulting in a slightly larger core size of 3.9699 Å (Figure S1, Supporting Information). However, the compressed core structure of the Cu_11_ABT NC likely results from reduced steric interactions among the outer ligands, promoting a more compact arrangement. Comparing the Cu_18_PTT NC with the reported [Cu_18_H_3_(SAdm)_12_(PPh_3_)_4_Cl_2_] (Cu_18_SAdm) (SAdm: adamantanethiolate) NC reveals distinct structural differences due to the variation in thiolate ligands (Figure S2, Supporting Information).^[^ ^19^ ^]^ While both NCs share a core‐shell architecture formed through a fusion process, the reported NC features the fusion of two distinct geometries, unlike the one discussed here. This highlights the critical role of thiolate ligands, reducing agents, and reaction conditions in precisely controlling NCs geometries.

### 2.3 Exploring Molecular Insights Through Characterization

**Figure 3:**
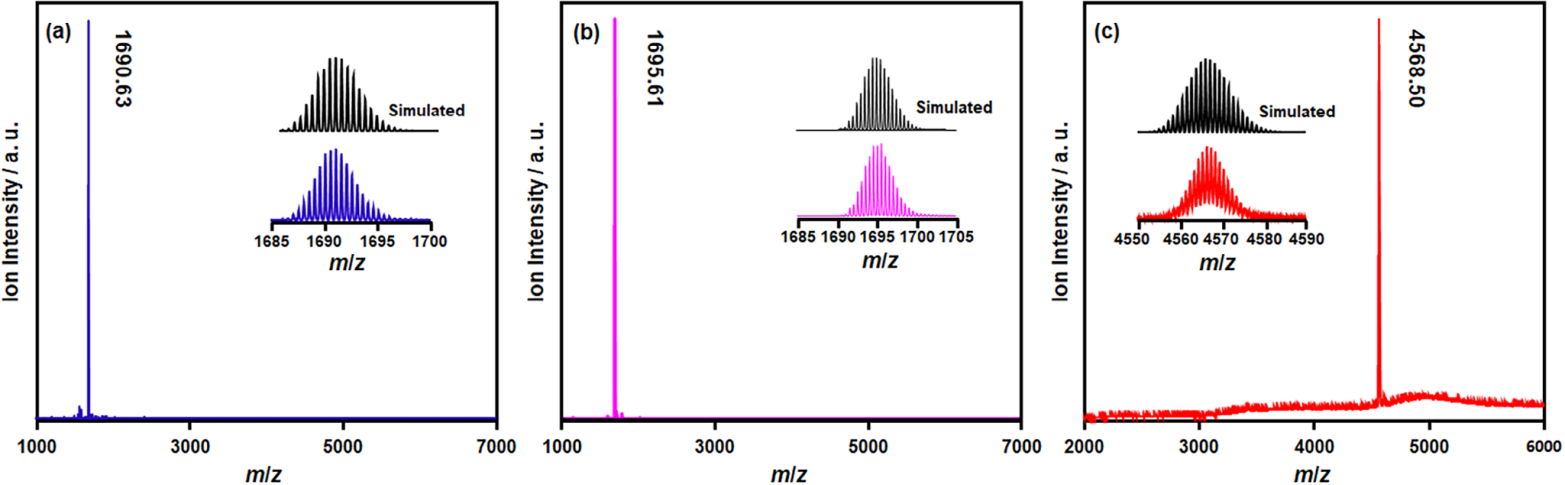
Positive mode ESI‐MS spectra of a) Cu_11_PTT NC, b) Cu_11_ABT NC, c) Cu_18_PTT NC. Insets show the matching of experimental and simulated peaks of molecular ions of the individual NCs with their respective charge states. Both the Cu_11_ NCs exhibit 2+ charge state and Cu_18_ NC exhibits neutral charge state.

**Figure 4:**
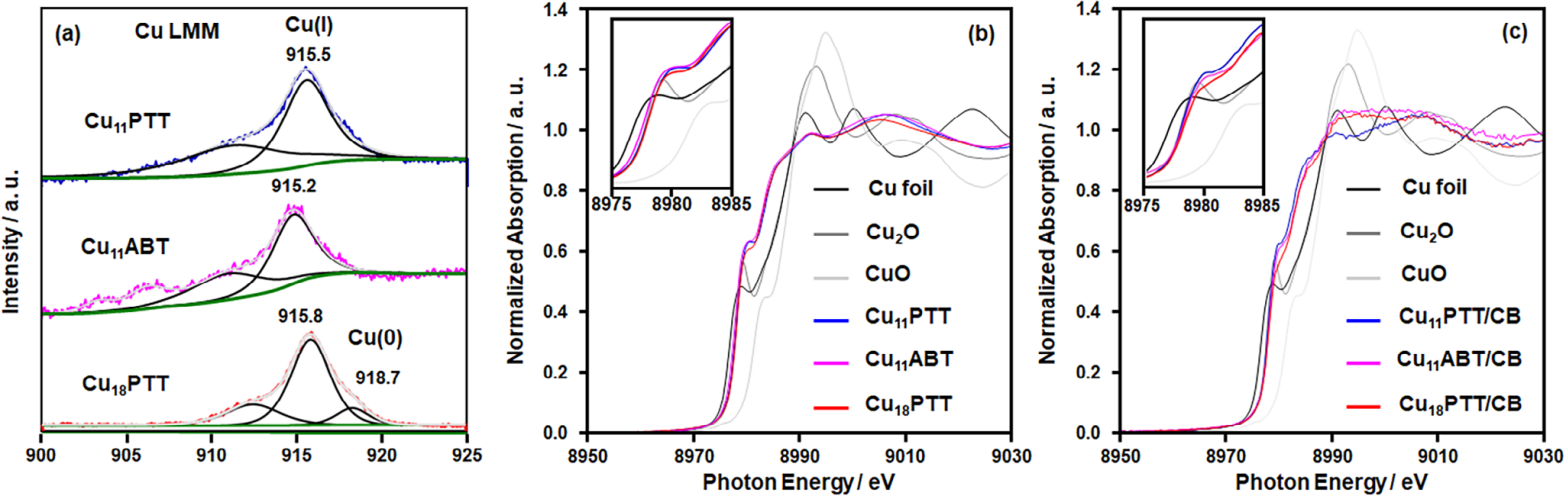
a) X‐ray excited Cu LMM Auger electron spectroscopy of all NCs, Cu‐K edge XANES spectra of b) all individual NCs and c) NCs loaded on CB. The slight difference in the XANES peak shape at ≈8995 eV is presumed to be due to the oxygen adsorption from air, but the *insitu* XANES spectra under CO_2_RR described below revealed that there was no significant change in the Cu NCs structure. Inset showing the zoomed region of the pre‐edge positions.

The identification of two hydrides in the Cu_18_PTT NC through SCXRD measurement suggests the preferred Cu atoms position to stabilize the hydrides in the NC unit which is not visible in the other two NCs. We then quantified the hydrides in Cu_18_PTT NC through the ESI‐MS measurements. In the positive mode ESI‐MS spectra, all the three NCs exhibit single molecular ion peak which suggests their robust stability. The peak at *m/z* = 1690.63 correspond to the [Cu_11_(PTT)_9_(PPh_3_)_6_]^2+^ (**Figure** **3**a) and peak at *m/z* = 1695.61 correspond to the [Cu_11_(ABT)_9_(PPh_3_)_6_]^2+^ (Figure 3b) matches well with their corresponding simulated spectra where the isotropic patterns confirm their divalent charge state. On the other hand, the peak at *m/z* = 4568.50 correspond to the simulated pattern of [[Cu_18_H_2_(PTT)_15_(PPh_3_)_6_] + H]^+^ (Figure 3c) indicating the fragmentation occurred during ionization via protonation. However, the origin of these hydrides is confirmed through the ESI‐MS analysis of a deuterated NC synthesized using NaBD_4_. The notable *m/z* shift of approximately +2 unequivocally indicates that the hydrides in the Cu_18_PTT NC are derived from the reducing agent (Figure S3, Supporting Information). In addition, the ESI‐MS experiment confirms the neutral charge state of Cu_18_PTT NC, indicating the presence of a single Cu(0) atom within the structure. X‐ray excited Cu LMM Auger electron spectroscopy (XAES) reveals peaks at 915.8 and 918.7 eV for the Cu_18_PTT NC, corresponding to Cu(I) and Cu(0), respectively, as determined from Gaussian–Lorentzian band fitting (**Figure** **4**a).^[^ ^20^ ^]^ The peak position at ≈911.0 eV in each spectrum confirms the elimination of the influence of other orbital electrons in the XAES. Notably, the peak at ≈918.7 eV, indicative of Cu(0), is absent in the two Cu_11_ NCs, confirming that Cu(0) is uniquely present in Cu_18_PTT NC. Furthermore, the X‐ray absorption near‐edge structure (XANES) spectra at the Cu K‐edge of the individual NCs reveal a distinct difference in the pre‐edge peak around ≈8980 eV between the Cu_11_ NCs and Cu_18_PTT NC. The slight lower intensity in the pre‐edge peak of Cu_18_PTT NC than Cu_11_ NCs, attributed to the presence of a mixed Cu(0)/Cu(I) oxidation state (Figure 4b). However, the Cu‐K edge Fourier transform‐extended X‐ray absorption fine structure (FT‐EXAFS) data confirms that there is no change in ligand coordination on the Cu. This is evident from the persistent peak observed at ≈1.8 Å, which is attributed to Cu─P or Cu─S bonding (Figure S4, Supporting Information). In addition, theoretical Bader charge analysis confirms that the Cu(0) atom is located at the center of the core (Figure S5, Supporting Information), bridging two Cu_5_ geometries, consistent with the previously reported structure of the Cu_18_SAdm NC. The bulk purity of these NCs is verified by the XPS survey spectra (Figure S6, Supporting Information).

### 2.4 Ensuring Integrity in the Catalyst Design Process

The effect of these three NCs is studied here by assessing their electrocatalytic activity studies. To perform the electrochemical measurements, thin film electrodes were initially fabricated for each NCs using a spray coating technique. This involved depositing a slurry of the individual NCs and carbon black (CB) onto a carbon paper substrate. It is crucial to maintain the geometric and electronic structure of the individual NCs during this loading process to preserve their inherent electrocatalytic properties. The stability of these NCs during this process was assessed using XAFS data. The similar pre‐edge peak position of XANES spectra at the Cu K‐edge for of loaded NCs with the individual NCs, indicating no notable changes in their electronic states (Figure 4c). Additional FT‐EXAFS spectra verified that the ligand environment remained intact during the loading process, as evidenced by a consistent peak at ∼1.8 Å, attributed to Cu─P or Cu─S bonds (Figure S7, Supporting Information). Moreover, TEM images further validated the discrete nature of each NC after being loaded onto the CB surface, showing that their sizes were preserved (Figures S8–S10, Supporting Information).

### 2.5 Electrocatalytic Reactivities and Their Impact on Product Selectivity

**Figure 5:**
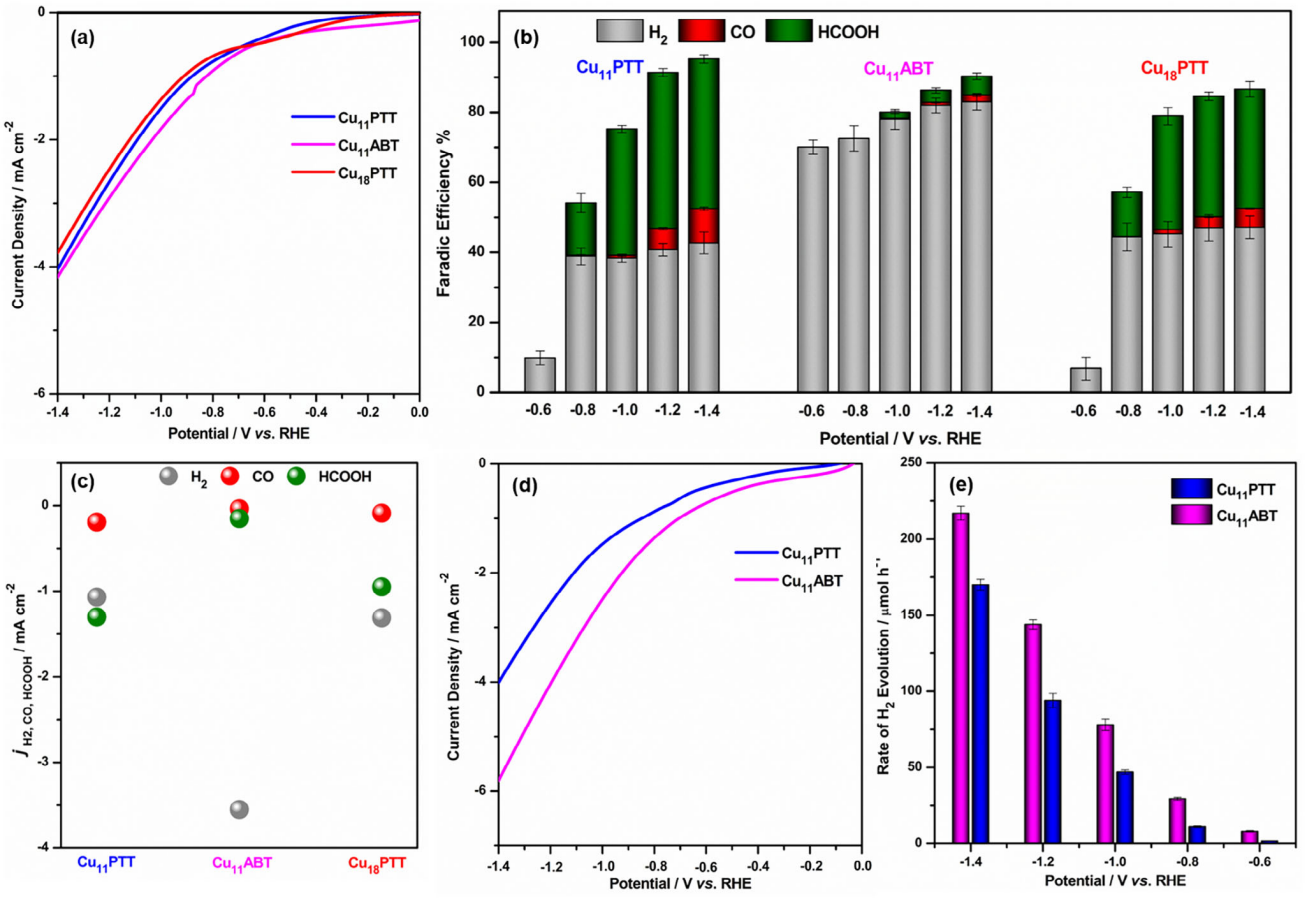
a) LSV b) FE (at different applied potential) and c) current density of the products (at −1.2 V vs RHE) in 0.1 m KHCO_3_ aq. under CO_2_ flow on Cu_11_PTT, Cu_11_ABT and Cu_18_PTT NCs containing catalyst. Error bars representing the mean ± SD, *n *= 3. d) LSV and e) rate of H_2_ evolution in a phosphate buffer medium at pH 7.4 for Cu_11_PTT and Cu_11_ABT NCs.

To assess the electrocatalytic activity of these NCs, we first investigated their CO₂RR capabilities using linear‐sweep voltammetry (LSV) in a CO₂‐saturated 0.1 m KHCO₃ aqueous electrolyte. The results showed that the Cu_11_ NCs exhibited the higher reduction currents, followed by the Cu_18_ NC (**Figure** **5**a). This initial finding underscores the potential of two Cu_11_ NCs with distinct ligands for electrocatalytic CO₂RR, even though one shares structural similarities in its ligand framework with the Cu_18_ NC. The detailed electrochemical performance of these NCs for CO₂RR was evaluated across a range of constant potentials (−0.6 to −1.4 V vs RHE) using an H‐type cell setup. The gaseous products were analyzed using gas chromatography (GC), while liquid‐phase products were characterized via ^1^H NMR spectroscopy (Figures S11–S13, Supporting Information). As shown in Figure 5b, the primary products were H_2_, CO, and HCOOH, but their selectivity was strongly influenced by the structural features of these NCs. To confirm the origin of the CO_2_RR products and ensure they were derived solely from the CO_2_ gas source, controlled experiments were conducted under an Ar gas flow in the absence of CO_2_. Under these conditions, H_2_ was the only detectable product, providing definitive evidence that the formation of CO and HCOOH observed during CO₂RR is solely attributable to the reduction of flowing CO₂, with no contributions from alternative sources (Figures S14–S16, Supporting Information). The Faradaic efficiency (FE) analysis of these NCs demonstrated significant variations in the FE values corresponding to the different products formed during the CO_2_RR (Figure 5b). These changes in FE were strongly influenced by the specific structural and compositional characteristics of each NC. It has been observed that, Cu₁₁PTT NC selectively produced HCOOH at potentials beyond −0.6 V (vs RHE), achieving a maximum FE_HCOOH_ of ≈45% at −1.2 V (vs RHE). In contrast, Cu₁₁ABT NC exhibited HER dominance throughout the potential range (−0.6 to −1.4 V vs RHE), with a maximum FE_H2_ of ≈83% at −1.4 V (vs RHE) (FE_H2_ of ≈82% at −1.2 V vs RHE). For Cu_11_ABT NC, the FE_HCOOH_ was substantially lower, peaking at only of ≈5% (FE_HCOOH_ of ≈3% at −1.2 V (vs RHE)) at the same potential. So, despite both NCs having the same number of Cu atoms and nearly identical overall geometries, they displayed stark differences in their electrocatalytic product selectivity and efficiency for CO_2_RR. Interestingly, Cu_18_PTT NC exhibited a distinct trend in the selectivity of CO_2_RR products. Although the Cu_18_PTT NC showed a modified core geometry compared to Cu_11_PTT NC, they still produced HCOOH, with the maximum FE_HCOOH_ reaching ≈35% at −1.2 V (vs RHE). The current density plots for the products formed at a potential of −1.2 V (vs RHE), further validate the observed trends in product distribution driven by the differing reactivities of these NCs (Figure 5c). Among these NCs, the Cu_11_PTT NC exhibits the highest current density for HCOOH production, followed by Cu_18_PTT and Cu_11_ABT NCs. So, it can be initially assumed that the superior role of surface ligands over structural architecture in governing CO_2_RR selectivity of HCOOH. A comparison of the reactivity between Cu_11_PTT and Cu_18_PTT NCs highlights the critical role of structural architecture in shaping catalytic activity. Notably, compared to our previous findings with [Cu_14_(SC_2_H_4_Ph)_3_(PPh_3_)_8_H_10_]^+^, [Cu_14_(SC_6_H_11_)_3_(PPh_3_)_8_H_10_]^+^, and [Cu_58_H_20_(SC_2_H_5_)_36_(PPh_3_)_6_]^2+^ NCs, the Cu₁₁PTT NC demonstrates superior selectivity for FE_HCOOH_.^[^ ^9^, ^14^ ^]^ This observation indicates that alterations in the geometric structure influence the configuration and properties of the active catalytic sites, thereby leading to variations in the FE of the products. However, the significant discrepancy in the CO_2_RR reactivity and product selectivity observed between the two Cu_11_ NCs—Cu_11_PTT and Cu_11_ABT—despite their identical geometry and number of Cu atoms, underscores the impact of surface ligand modification. Thus, these observations underscore the interplay between surface ligands and geometric structure in determining the electrocatalytic performance of Cu NCs.

To gain deeper insight into the ligand effect, the difference in HER activity between the two Cu_11_ NCs is analyzed using LSV measurements in a phosphate buffer medium at pH 7.4. Interestingly, the Cu_11_ABT NC exhibited a higher current density compared to Cu_11_PTT NC, indicating greater efficiency of Cu_11_ABT NC toward the HER (Figure 5d). Moreover, at any fixed overpotentials, the mass‐based H_2_ evolution activity of Cu_11_ABT NC was significantly superior to that of Cu₁₁PTT NC (Figure 5e). To understand the underlying reason for this observation, we hypothesized that the variation in HER activity might be attributed to differences in hydrophilicity stemming from the distinct ligand systems of the NCs. We observed that Cu_11_ABT NC exhibits significantly different solubility in polar protic solvents compared to Cu_11_PTT NC, attributed to their hydrophilic surface characteristics (Figure S17, Supporting Information). However, this difference does not fully represent the actual scenario when a catalytic amount of these NCs is loaded onto the hydrophobic CB surface and subsequently coated onto the hydrophobic carbon paper substrate. To better understand the real behavior, we performed a drop test on carbon paper coated with the respective catalysts (Figure S18, Supporting Information). The results revealed that a water droplet collapsed rapidly on the Cu_11_ABT NC‐coated sample, indicating its higher hydrophilicity, whereas on the Cu_11_PTT NC‐coated sample, the droplet remained intact for an extended period, suggesting a more hydrophobic surface.

The observed difference in surface hydrophilicity is likely to play a critical role in determining the product selectivity during the CO₂RR, primarily by influencing the proton diffusion and adsorption processes. The hydrophilic nature of the outer surface of Cu₁₁ABT NCs facilitates enhanced proton transport by promoting the proton‐jumping mechanism.^[^ ^21^ ^]^ This efficient proton mobility favors the HER, thereby influencing the competing CO_2_RR pathway. In contrast, the hydrophobic surface characteristics of Cu_11_PTT NC relatively hinder the proton diffusion and adsorption. This deceleration in proton transport dynamics reduces the propensity for HER, thereby shifting the reaction selectivity more significantly toward competing HCOOH formation under a continuous flow of CO_2_.

### 2.6 Mechanistic Insights into the Different Reactivities

**Figure 6:**
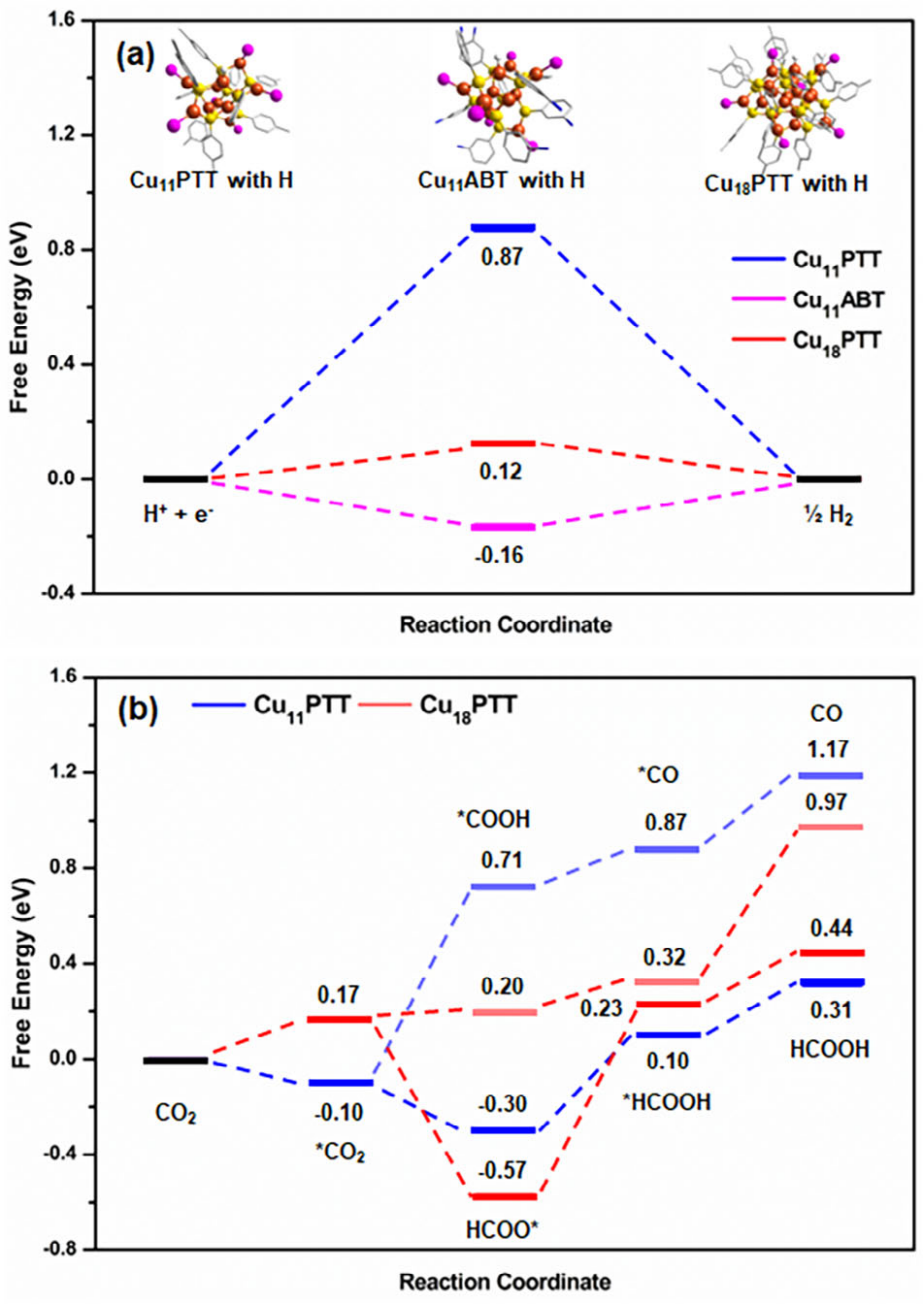
Free energy diagram and corresponding intermediates a) HER inset showing the individual NCs with H adsorption sites and b) CO_2_RR.

The performance of these NCs in electrochemical CO_2_RR shows remarkable variation despite similarities in their structural architecture or thiolate‐capped ligand surfaces. The disparity in selectivity and efficiency of CO_2_RR products is assumed to stem from differences in the proton transfer mechanisms inherent to these NCs. Notably, neither structure contains lattice hydride, thereby precluding the straightforward utilization of hydrides during the reduction process. This absence necessitates an alternative hydrogen acquisition pathway, where the generated hydrogen must be successfully adsorbed on the cluster surface prior to transfer. To investigate the reaction mechanism and the origin of the selectivity differences among the NCs, density functional theory (DFT) calculations are performed using a simplified cluster system (see details in Figure S19, Supporting Information). The theoretical analysis highlights that the free energy change for *H adsorption is lowest (−0.16 eV) for Cu_11_ABT NC compared to Cu_11_PTT and Cu_18_PTT NCs, consistent with this pronounced preference for the HER (**Figure** **6**a). The difference in *H adsorption energy between Cu_11_ NCs variants can be attributed to the unique *H adsorption sites dictated by its confined structural architectures and stronger electronic interactions with favorable localized electron density. This structural confinement optimizes the overlap between the active Cu sites and *H intermediates, reducing the HER energy barrier and driving the reaction efficiently. This trend continues with Cu_18_PTT NC, where the level of confinement is reduced, yet remains present despite differences in structural organization (Figure 6a). Thus, the experimental HER, which serves as the primary competitive side reaction during CO_2_RR, follows the order: Cu_11_ABT > Cu_18_PTT > Cu_11_PTT NC which is also consistent with the computational results. To further elucidate the differences in CO_2_RR efficiency and selectivity between Cu_11_PTT NC and Cu_18_PTT NC, we explored two dominant reaction pathways: one leading to HCOOH and the other to CO. The variation in structural architecture impacts CO_2_ adsorption sites, influencing the energy profiles of the pathways (Figure 6b). For both NCs, the energy diagrams favor the production of HCOOH over CO, which is driven by stronger intermediate stabilization and favorable lower limiting potentials of HCOO* to *HCOOH than the *COOH to *CO. Specifically, Cu_11_PTT NC shows a significantly lower free energy (−0.57 eV) for *HCOO formation compared to Cu_18_PTT NC (−0.30 eV), highlighting its superior thermodynamic preference for HCOOH production. The significant stabilization of the *HCOO intermediate on Cu_11_PTT NC arises from its optimized active sites and its core atomic framework. Conversely, Cu_18_PTT NC demonstrates a stronger preference for competitive side reaction than Cu_11_PTT NC. This is evidenced by its lower energy barrier for H adsorption, which competes directly with CO_2_RR intermediates. To further validate these findings, d‐band center energy calculations were performed, which indicate higher reactivity for Cu_11_PTT NC over Cu_18_PTT NC (Figure S20, Supporting Information). The d‐band center analysis reveals that Cu_11_PTT NC has a d‐band center closer to the Fermi level, exhibiting stronger interaction with CO_2_RR intermediates compared to Cu_18_PTT NC. These differences in reactivity and selectivity, despite both NCs sharing similar thiolate ligands on their surfaces, underscore the pivotal role of structural architecture in dictating product distribution and efficiency for both CO_2_RR and the competitive HER. Ultimately, the interplay between structural confinement, electronic properties, and ligand environments drives the catalytic performance of the NCs. While the ligand environment influences the outer surface interactions, the unique spatial arrangement of the atomic framework also governs the adsorption dynamics, reaction pathways, and product distribution.

### 2.7 Sustainability of these NCs over Time

To assess the stability of these NCs under applied electrochemical conditions and their catalytic effectiveness, chronoamperometric measurements were conducted at −1.2 V (vs RHE) over 12 h (Figure S21, Supporting Information). The results for Cu_11_PTT NC reveal that the total FE gradually increases, and remains stable thereafter. The rise in total FE is largely attributed to contributions from all three products (FE_HCOOH_ reaches ≈46%), with the HER being the dominant factor. A quite similar upward trend in FE was observed for the other two NCs. We assume that this improvement in total FE is associated with an increase in the reactivity of the NCs, likely due to the partial loss of ligands during prolonged electrochemical measurements. However, Cu K‐edge *insitu* XANES spectra revealed no notable alterations in spectral features compared to each as synthesized NC, indicating the structural stability of the NCs (Figure S22, Supporting Information). This was further corroborated by *insitu* FT‐EXAFS spectra, which confirmed the stability of Cu─S or Cu─P bonds under electrochemical conditions (Figure S22, Supporting Information). To further evaluate the stability of these NCs outside the electrochemical environment post‐electrocatalysis, TEM imaging demonstrated the structural integrity of the clusters, as evidenced by their consistent size (Figure S23, Supporting Information). However, FT‐IR analysis revealed a persistent P═O peak, which is likely due to the loss of neutral PPh_3_ ligands from the NCs (Figure S24, Supporting Information). This loss of neutral ligands does not significantly alter the electronic environment of the NCs, preserving their stability. Notably, the removal of bulky ligands from the NC surface enhances the reactivity of these NCs under electrochemical conditions, resulting in an increased total FE after a certain period. Once this initial ligand loss stabilizes, the NCs maintain their reactivity and total FE over extended durations, consistent with their performance after 10 h.

## 3 Conclusion

In summary, we have demonstrated that precise control over the reaction conditions during nanocluster (NC) synthesis allows for fine‐tuning of the structural architecture, which significantly impacts the catalytic properties of a series of Cu NCs. Our findings underscore that subtle variations in reaction parameters validate the critical role of surface ligands in determining the overall geometry while highlighting the influence of core confinement in governing catalytic behavior. Specifically, we observed that the non‐confined Cu_5_ core in Cu_11_PTT NC exhibits superior selectivity for HCOOH during electrochemical CO_2_RR. In contrast, the Cu_18_ NC, which features a larger but more confined core structure and a similar ligand environment, demonstrated reduced selectivity for HCOOH under identical reaction conditions. This outcome highlights core confinement as a decisive factor influencing product selectivity. Furthermore, when the hydrophobic surface ligands were replaced with hydrophilic ligands, coupled with enhanced core confinement, a notable shift toward competing side reactions was observed. Thus, this study reveals a complex interplay between core geometry, core confinement, and surface ligand properties in dictating the catalytic performance of Cu NCs. These insights not only deepen our understanding of structure‐activity relationships in NCs but also pave the way for the rational design of next‐generation catalysts with tailored selectivity and efficiency for electrochemical CO_2_RR and related catalytic processes.

## Conflict of Interest

The authors declare no conflict of interest.

## Supporting information



Supporting Information

## Data Availability

The data that support the findings of this study are available from the corresponding author upon reasonable request.

## References

[1] a) S. J. Davis , K. Caldeira , H. D. Matthews , Science 2010, 329, 1330; 20829483 10.1126/science.1188566

[2] a) Y. J. Sa , C. W. Lee , S. Y. Lee , J. Na , U. Lee , Y. J. Hwang , Chem. Soc. Rev. 2020, 49, 6632; 32780048 10.1039/d0cs00030b

[3] a) F. Franco , C. Rettenmaier , H. S. Jeon , B. R. Cuenya , Chem. Soc. Rev. 2020, 49, 6884; 32840269 10.1039/d0cs00835d

[4] a) N. W. Kinzel , C. Werlé , W. Leitner , Angew. Chem., Int. Ed. 2021, 60, 11628; 10.1002/anie.202006988 PMC8248444 33464678

[5] a) S. Liang , L. Huang , Y. Gao , Q. Wang , B. Liu , Adv. Sci. 2021, 8, 2102886; 10.1002/advs.202102886 PMC8693035 34719862

[6] a) D. Yang , J. Wang , Q. Wang , Z. Yuan , Y. Dai , C. Zhou , X. Wan , Q. Zhang , Y. Yang , ACS Nano 2022, 16, 15681; 36121680 10.1021/acsnano.2c06059

[7] a) G. Ma , L. Qin , Y. Liu , H. Fan , L. Qiao , C. Yu , Z. Tang , Surf. Interfaces 2023, 36, 102555;

[8] a) D. R. Kauffman , D. Alfonso , C. Matranga , H. Qian , R. Jin , J. Am. Chem. Soc. 2012, 134, 10237; 22616945 10.1021/ja303259q

[9] a) S. Biswas , S. Das , Y. Negishi , Nanoscale Horiz. 2023, 8, 1509; 37772632 10.1039/d3nh00336a

[10] Q. Tang , Y. Lee , D.‐Y. Li , W. Choi , C. Liu , D. Lee , D.‐e. Jiang , J. Am. Chem. Soc. 2017, 139, 9728. 28640611 10.1021/jacs.7b05591

[11] F. Li , Q. Tang , J. Catal. 2020, 387, 95.

[12] L. J. Liu , Z. Y. Wang , Z. Y. Wang , R. Wang , S. Q. Zang , T. C. Mak , Angew. Chem., Int. Ed. 2022, 61, 202205626. 10.1002/anie.202205626 35672885

[13] Q. J. Wu , D. H. Si , P. P. Sun , Y. L. Dong , S. Zheng , Q. Chen , S. H. Ye , D. Sun , R. Cao , Y. B. Huang , Angew. Chem., Int. Ed. 2023, 62, 202306822. 10.1002/anie.202306822 37468435

[14] S. Biswas , T. Tanaka , H. Song , M. Ogami , Y. Shingyouchi , S. Hossian , M. Kamiyama , T. Kosaka , R. Nakatani , Y. Niihori , S. Das , T. Kawawaki , D.‐e. Jiang , Y. Negishi , Small Sci. 2025, 5, 2400465. 40213063 10.1002/smsc.202400465 PMC11934906

[15] a) S. Biswas , Y. Negishi , Dalton Transact. 2024, 53, 9657; 10.1039/d4dt00296b 38624154

[16] M. Kamiyama , Y. Shingyouchi , R. Sarma , M. Ghosh , T. Kawawaki , S. Biswas , Y. Negishi , Chem. Commun. 2025, 61, 1048. 10.1039/d4cc06139j 39660545

[17] a) G. G. Luo , Z. H. Pan , B. L. Han , G. L. Dong , C. L. Deng , M. Azam , Y. W. Tao , J. He , C. F. Sun , D. Sun , Angew. Chem., Int. Ed. 2023, 62, 202306849; 10.1002/anie.202306849 37469101

[18] H. Li , H. Zhai , C. Zhou , Y. Song , F. Ke , W. W. Xu , M. Zhu , J. Phys. Chem. Lett. 2020, 11, 4891. 32490675 10.1021/acs.jpclett.0c01358

[19] A. K. Das , S. Biswas , V. S. Wani , A. S. Nair , B. Pathak , S. Mandal , Chem. Sci. 2022, 13, 7616. 35872832 10.1039/d2sc02544b PMC9241973

[20] A. Wang , M. Zhang , H. Yin , S. Liu , M. Liu , T. Hu , RSC Adv. 2018, 8, 19317. 35539692 10.1039/c8ra03125h PMC9080717

[21] a) K. Kwak , W. Choi , Q. Tang , D.‐e. Jiang , D. Lee , J. Mater. Chem. A 2018, 6, 19495;

