# Supplementary material for: Deciphering Electrocatalytic Activity in Cu Nanoclusters: Interplay Between Structural Confinement and Ligands Environment
Source: Small. 2025 Mar 6;21(25):2500302. doi: 10.1002/smll.202500302 (PMC12199118; doi:10.1002/smll.202500302)
Supplement: Supplementary file 1 — Supporting Information [file SMLL-21-2500302-s001.docx]

**Supporting Information**

**Deciphering Electrocatalytic Activity in Cu Nanoclusters: Interplay between Structural Confinement and Ligands Environment**

Sourav Biswas,^[a]ϯ^ Yamato Shingyouchi,^[b]ϯ^ Maho Kamiyama,^[b]^ Milan Kumar Jena,^[c]^ Masaki Ogami,^[b]^ Tokuhisa Kawawaki,* ^[a,b]^ Biswarup Pathak,* ^[c]^ and Yuichi Negishi*^[a,d]^

^a^ Research Institute for Science & Technology, Tokyo University of Science, 1-3 Kagurazaka, Shinjuku-ku, Tokyo 162-8601, Japan.

^b^ Department of Applied Chemistry, Faculty of Science, Tokyo University of Science, 1-3 Kagurazaka, Shinjuku-ku, Tokyo 162-8601, Japan.

^c^ Department of Chemistry, Indian Institute of Technology Indore, Indore, Madhya Pradesh 453552, India.

^d^ Institute of Multidisciplinary Research for Advanced Materials, Tohoku University, Katahira 2-1-1, Aoba-ku, Sendai 980-8577, Japan.

Ϯ Equal contributions

*Corresponding Authors

T.K.: kawawaki@rs.tus.ac.jp

B.P.: biswarup@iiti.ac.in

Y.N.: negishi@rs.tus.ac.jp

**Table of Contents**

| **Name** | **Description** | **Page No.** |
| --- | --- | --- |
|  | Experimental section | S3-S9 |
| Table S1 | Crystal data and structure refinement parameters of Cu_11_PTT NC | S10 |
| Table S2 | Crystal data and structure refinement parameters of Cu_11_ABT NC | S11 |
| Table S3 | Crystal data and structure refinement parameters of Cu_18_PTT NC | S12 |
| Figure S1 | Structural architecture of the reported Cu_11_TBBT NC | S13 |
| Figure S2 | Structural architecture of the reported Cu_18_SAdm NC | S13 |
| Figure S3 | Positive mode ESI-MS spectra of [Cu_18_D_2_(PTT)_15_(PPh_3_)_6_] NC | S13 |
| Figure S4 | Cu-K edge FT-EXAFS data of all Cu NCs | S14 |
| Figure S5 | Badar charge analysis of individual Cu atoms present in Cu_18_PTT NC | S14 |
| Figure S6 | XPS survey spectrum of all NCs | S15 |
| Figure S7 | FT-EXAFS data of loaded NCs on CB surface | S16 |
| Figure S8 | TEM images of Cu_11_PTT NCs and Cu_11_PTT loaded on CB | S16 |
| Figure S9 | TEM images of Cu_11_ABT NCs and Cu_11_ABT loaded on CB | S17 |
| Figure S10 | TEM images of Cu_18_PTT NCs and Cu_18_PTT loaded on CB | S17 |
| Figure S11 | The raw data for CO_2_RR results under CO_2_ flow on Cu_11_PTT NC loaded catalysts | S18 |
| Figure S12 | The raw data for CO_2_RR results under CO_2_ flow on Cu_11_ABT NC loaded catalysts | S18 |
| Figure S13 | The raw data for CO_2_RR results under CO_2_ flow on Cu_18_PTT NC loaded catalysts | S18 |
| Figure S14 | The raw data for CO_2_RR results under Ar flow on Cu_11_PTT NC loaded catalysts | S19 |
| Figure S15 | The raw data for CO_2_RR results under Ar flow on Cu_11_ABT NC loaded catalysts | S19 |
| Figure S16 | The raw data for CO_2_RR results under Ar flow on Cu_18_PTT NC loaded catalysts | S19 |
| Figure S17 | Solubility test for Cu_11_ NCs | S20 |
| Figure S18 | Drop-test on carbon paper loaded specific NC with water droplet | S20 |
| Figure S19 | Theoretically optimized structures | S20 |
| Figure S20 | d-band center relative to the Fermi level for Cu_11_PTT and Cu_18_PTT NCs | S20 |
| Figure S21 | Long term chronoamperometric measurements of all Cu NCs | S21 |
| Figure S22 | Cu-K edge *insitu* XANES and *insitu* FT-EXAFS data of all Cu NCs after CO_2_RR | S21 |
| Figure S23 | TEM images of the individual Cu NCs catalysts after CO_2_RR | S22 |
| Figure S24 | FT-IR of individual NCs and catalysts before and after CO_2_RR | S23 |
|  | References | S24 |

**Experimental Section:**

**Reagents**

Tetrakis(acetonitrile)copper(I) tetrafluoroborate Cu(CH_3_CN)_4_(BF_4_), triphenylphosphine (PPh_3_), *p*-toluenethiol (PTT), *m*-aminobenzenethiol (ABT), sodium borohydride (NaBH_4_) were procured from TCI. Borane *tert*-butylamine complex and sodium borodeuteride (NaBD_4_) were procured from Sigma Aldrich. HPLC grade solvents- chloroform, methanol, acetonitrile, dimethyl sulfoxide and n-hexane were purchased from TCI. Water, 2-propanol and Nafion^®^ were procured from FUJIFILM Wako Pure Chemical Corporation. Potassium bicarbonate, phosphate buffer was procured from Kanto Chemical Co., Inc. Carbon black (Vulcan XC-72) was procured from Fuel Cell Earth and Carbon paper (SIGRACET®GDL 36BB) was procured from SGL carbon.

Materials characterization

Electrospray ionization mass spectrometry was performed using a reflectron-type time of flight MS system (Bruker, microTOF II). The Cu_11_PTT, Cu_11_ABT and Cu_18_PTT NCs were dissolved in a mixture of dichloromethane and methanol. The isotope distribution was calculated using an isotope pattern simulator (JEOL, Isotope Pattern Simulator). ICP-MS was performed with an Agilent 7850c spectrometer (Agilent Technologies, Tokyo, Japan). Bi was used as the internal standard. Cu standard solution were used for drawing calibration line. The ICP-MS measurements were performed for the solution before mixing Cu_11_PTT, Cu_11_ABT and Cu_18_PTT NCs with CB to estimate the adsorbed or loaded Cu content. The X-ray photoelectron spectroscopy (XPS) spectra were collected by using a JPS-9010MC electron spectrometer (JEOL, Tokyo, Japan) at a base pressure of ∼2 × 10^−8^ Torr. X-rays from the Mg-Kα line (1253.6 eV) were used for excitation. Each NCs was deposited on an In_2_O_3_ plate and the spectra were referenced to the trivalent In 3d 5/2 peak at 444.6 eV. The transmission electron microscope (TEM) images were recorded with a JEM-2100 electron microscope (JEOL, Tokyo, Japan) operating at 200 kV, typically using magnification of 600,000. X-ray absorption fine structure (XAFS) measurements were performed at beamline BL01B1 of the SPring-8 facility of the Japan Synchrotron Radiation Research Institute (proposal numbers 2023B1825 and 2024A1698, 2024B1592). The incident X-ray beam was monochromatized with a Si(111) double-crystal monochromator. Cu K-edges of XAFS spectra of all samples (as well as Cu foil, Cu_2_O powder, and CuO powder as a reference) were recorded in transmission mode with ionization chambers. The X-ray energies for the Cu K-edges were calibrated with Cu foil, respectively. X-ray absorption near-edge structure (XANES) and extended XAFS (EXAFS) spectra were analyzed with xTunes^S1^ as follows. The χ spectra were extracted by subtracting the atomic absorption background by cubic spline interpolation and normalized to the edge height. The normalized data were used as the XANES spectra. The k^3^-weighted χ spectra in the k range 3.0–12.0 Å^−1^ for the Cu K-edges were Fourier-transformed into r space for structural analysis. FT-IR spectra were obtained using the attenuated total reflectance (ATR) method in the region of 400 and 4000 cm_−1_ by a FT/IR-4600-ATR-PRO ONE spectrometer (JASCO, Tokyo, Japan) equipped with a DLATGS detector as the average of 500 scans at 4 cm_−1_ resolution.

Synthesis of Cu_11_PTT NC

The entire reaction process was carried out in a 50 mL vial at ambient condition. First, commercially available (Cu(CH_3_CN)_4_BF_4_, 160 mg, 0.509 mmol was dissolved by adding acetonitrile (10 mL). Then, 130 mg of PPh_3_ was added to the reaction mixture and kept stirring until dissolving PPh_3_ for 10 minutes. After fully dissolving PPh_3_ powder in solution, 36.8 mg of PTT (in 200 µL of acetonitrile) and kept stirring until dissolving PPh_3_ for 30 minutes. Then, 200 mg of Borane *tert*-butylamine complex (in 3 mL of methanol) and 3 mL of acetone was quickly added in order. The color of the solution immediately changed from clear to orange. The reaction mixture was stirred for 3 hours. Subsequently, the solvent was removed with a rotary evaporator, and the residue was dissolved in 10 mL of methanol. The mouth of the vial was sealed and allowed to stand at dark place for 1 day to obtain the [Cu_11_(PTT)_9_(PPh_3_)_6_]^2+^, as yellow colored, crystals.

Synthesis of Cu_11_ABT NC

The entire reaction process was carried out in a 50 mL vial at ambient condition as previous. First, commercially available (Cu(CH_3_CN)_4_BF_4_), 160 mg, 0.509 mmol was dissolved by adding acetonitrile (4 mL) and chloroform (4 mL) in that order. Then, 130 mg of PPh_3_ was added to the reaction mixture and kept stirring until dissolving PPh_3_ for 10 minutes. After fully dissolving PPh_3_ powder in solution, 20 µL of ABT was added. The color of the solution changed from clear to yellow. After confirming that the color no longer changes, 30 mg of NaBH_4_ (in 3 mL of methanol) was quickly added. The color of the solution immediately changed from yellow to reddish brown. The reaction mixture was stirred for 3 hours. Subsequently, the solvent was removed with a rotary evaporator, and the residue was dissolved in 10 mL of methanol. The mouth of the vial was sealed and allowed to stand at dark place for 2 days to obtain the [Cu_11_(ABT)_9_(PPh_3_)_6_]^2+^, as yellow colored, crystals.

Synthesis of Cu_18_PTT NC

The entire reaction process was carried out in a 50 mL vial in an ice bath. First, commercially available (Cu(CH_3_CN)_4_BF_4_), 160 mg, 0.509 mmol was dissolved by adding acetonitrile (4 mL) and chloroform (4 mL) in that order, 130 mg of PPh_3_ was added to the reaction mixture and kept stirring until dissolving PPh_3_ for 10 minutes. After fully dissolving PPh_3_ powder in solution, 36.8 mg of PTT (in 200 µL of acetonitrile). Then, 30 mg of NaBH_4_ (in 3 mL of methanol) was quickly added. The color of the solution immediately changed from clear to yellow. The reaction mixture was stirred for 5 hours. Subsequently, the solvent was removed with a rotary evaporator, and the residue was washed twice with 10 mL of methanol and dissolved in 10 mL of chloroform. From the upper side of the obtained solution, 10 mL of hexane was poured gently and layered on the top of the chloroform solution. The mouth of the vial was sealed and allowed to stand in a refrigerator for 1 month to obtain the [Cu_18_H_2_(PTT)_15_(PPh_3_)_6_], as yellow colored, crystals.

Methods of Density Functional Theory (DFT) calculations

The electric structure calculation of three considered NCs, Cu_11_PTT, Cu_11_ABT, and Cu_18_PTT, was performed using density functional theory (DFT)^S2^ as implemented in the Vienna Ab initio Simulation Package (VASP) code.^S3^ The crystal structures of three NCs were considered as the initial geometry during structural optimization. For the sake of simplicity, the PPh_3_ moiety was replaced with PH_3_ during the calculations. The electron-ion interactions were treated with the projector-augmented plane-wave (PAW) method.^S4^ The Generalized Gradient Approximation of Perdew−Burke−Ernzerhof (GGA-PBE) was employed to model the exchange-correlation interactions.^S5-6^ The van der Waals interactions were included using the empirical correction in Grimme’s scheme (DFT-D3).^S7^ A plane-wave cutoff of 470 eV was set for all the computations in this study. The Brillouin zone was sampled using only Γ point. The energy convergence criterion and Hellmann-Feynman forces acting on atoms converged below 10^–5^ eV per atom and smaller than 0.05 eV Å^–1^, respectively. The NCs were placed in a cubic box with dimensions of 26 × 26 × 26 Å^3^, which is sufficient to avoid any periodic image interactions. All atoms were unconstrained and fully relaxed during the simulation. The reaction free energies of the electrochemical steps were calculated using the computational hydrogen electrode proposed by Nørskov et al.^S8^ Gibbs free energies ($\boldsymbol{\Delta G}$) for all the electrochemical or non-electrochemical steps were computed by the following equation:

$$\boldsymbol{\Delta G = \Delta E +}\boldsymbol{\Delta E}_{\boldsymbol{zpe}}\boldsymbol{- T\Delta S}$$

where $\boldsymbol{\Delta E}$ is the difference of the DFT total energy, $\boldsymbol{\Delta E}_{\boldsymbol{zpe}}$ is the zero-point energy difference calculated from the vibrational frequencies, and $\boldsymbol{\Delta S}$ is the entropy difference between the products and the reactants. The entropies of the free molecules at 298 K and 1 atm were taken from the NIST database, while the vibrational entropy was computed for the adsorbed species.

Catalyst preparation


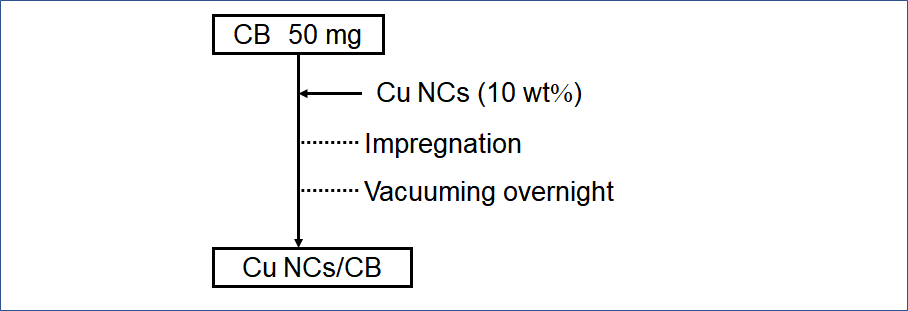
To prepare Cu NCs loaded electrocatalysts (Cu_11_PTT NC/CB, Cu_11_ABT NC/CB, Cu_18_PTT NC/CB), Cu NCs were loaded onto the CB using the impregnation method (Scheme S1). Specifically, Cu_11_PTT and Cu_18_PTT NCs crystal were dissolved in chloroform, and Cu_11_ABT NC crystal were dissolved in dichloromethane. The concentration was measured by ICP-MS. Then the NC solution was added to the CB. In this process, the Cu loading ratio was set to 10 wt%. The mixture was mixed at room temperature until the solvent evaporated. Finally, Cu NCs/CB was obtained by evacuating overnight in a desiccator.

Scheme S1. Preparation method of Cu NCs/CB. A similar method was used to prepare Cu_11_PTT/CB, Cu_11_ABT/CB and Cu_18_PTT/CB.

Electrochemical measurement for the CO_2_RR

All electrochemical measurements for the CO_2_RR were performed with an ECstat-302 (EC FRONTIER, Japan) with a flow cell (EC FRONTIER, Japan). First, to prepare the catalyst slurry, Cu catalyst (12 mg) was added to a solution consisting of ultrapure water (2 mL), 2-propanol (0.5 mL), and Nafion^®^ solution (10 μL). The obtained mixture was sonicated in an ice-water bath for 30 min to disperse the Cu catalyst (Cu_11_PTT/CB, Cu_11_ABT/CB and prepare Cu_18_PTT/CB) and afford a catalyst slurry. Then, the catalyst slurry (1.1 mL) was sprayed on carbon paper (SIGRACET^®^GDL 36BB) (φ = 2 cm), which was used as the working electrode. A Pt mesh electrode was used as the counter electrode. A silver/silver chloride (Ag/AgCl) electrode was used as the reference electrode. Each electrode was set in an electrochemical measurement system containing 0.1 M KHCO_3_ (pH = ~7 under CO_2_ sat.) as the electrolyte. In the measurements, CO_2_ gas was bubbled for 15 min and then CV was conducted for cleaning the electrodes. After CV, CA was performed under CO_2_ (flow rate: 10 mL min^–1^) at –0.6 V (vs. RHE) for 1 h. After that, the electrolyte is replaced and CO_2_ bubbling is performed for the next CA. The detailed scheme is shown in Scheme S2. Gas products were analyzed by online gas chromatograph (Shimadzu, GC-8A, TCD or FID; Ar or N_2_ carrier gas, respectively). Liquid products were analyzed by ^1^H NMR spectroscopy. Typically, 590 μL of electrolyte after
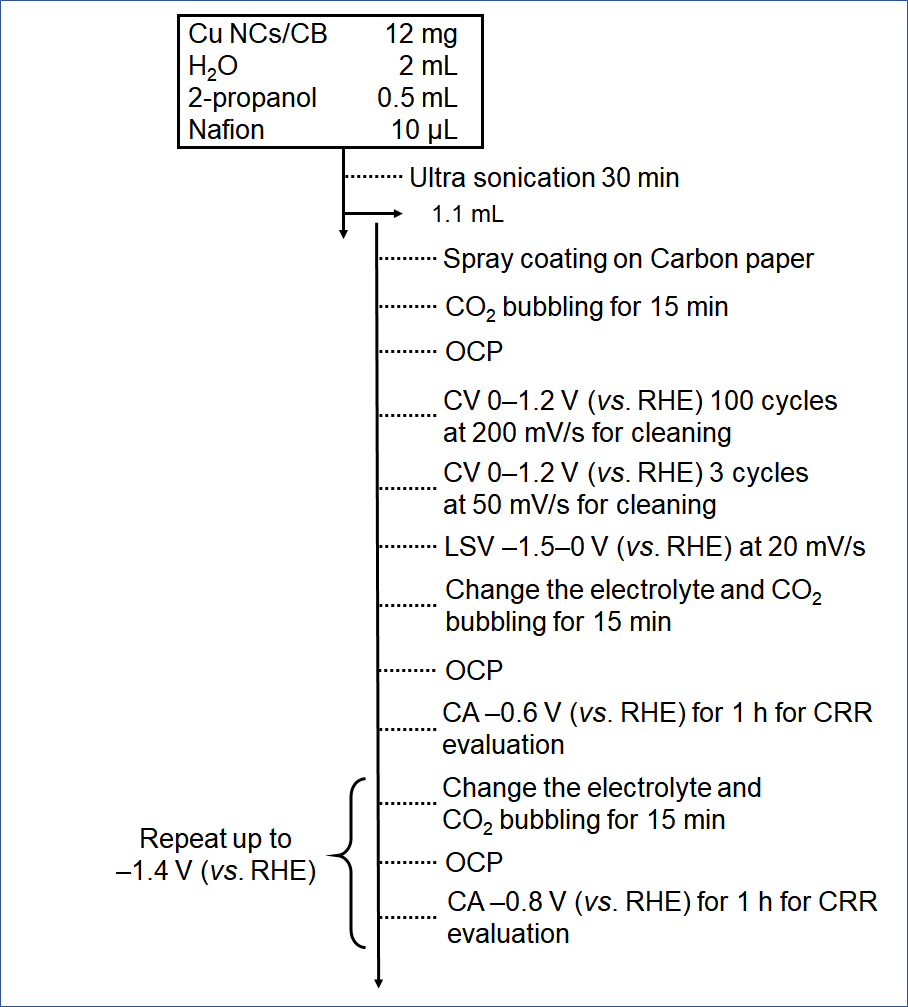
electrolysis was mixed with 10 μL of DMSO as internal standard.

Scheme S2. Preparation method of working electrode and electrochemical measurement protocol for CO_2_RR.

Electrochemical measurement for the HER

All electrochemical measurements for the HER were performed with an ECstat-302 (EC FRONTIER, Japan) with a flow cell (EC FRONTIER, Japan). First, to prepare the catalyst slurry, Cu catalyst (12 mg) was added to a solution consisting of ultrapure water (2 mL), 2-propanol (0.5 mL), and Nafion^®^ solution (10 μL). The obtained mixture was sonicated in an ice-water bath for 30 min to disperse the Cu catalyst (Cu_11_PTT/CB, Cu_11_ABT/CB) and afford a catalyst slurry. Then, the catalyst slurry (1.1 mL) was sprayed on carbon paper (SIGRACET^®^GDL 36BB) (φ = 2 cm), which was used as the working electrode. A Pt mesh electrode was used as the counter electrode. A silver/silver chloride (Ag/AgCl) electrode was used as the reference electrode. Each electrode was set in an electrochemical measurement system containing phosphate buffer (pH = ~7.4 under Ar sat.) as the electrolyte. In the measurements, Ar gas was bubbled for 15 min and then cyclic voltammetry (CV) was conducted for cleaning the electrodes. After CV, Chronoamperometry (CA) was performed under Ar (flow rate: 10 mL min^–1^) at –0.6 V (vs. RHE) for 1 h. After that, the electrolyte is replaced and Ar bubbling is performed for the next CA. The detailed scheme is shown in Scheme S3. Gas products were analyzed by online gas chromatograph (Shimadzu, GC-8A, TCD or FID; Ar or N_2_ carrier gas, respectively). Liquid products were analyzed by ^1^H NMR spectroscopy. Typically, 590 μL of electrolyte after electrolysis was mixed with 10 μL of
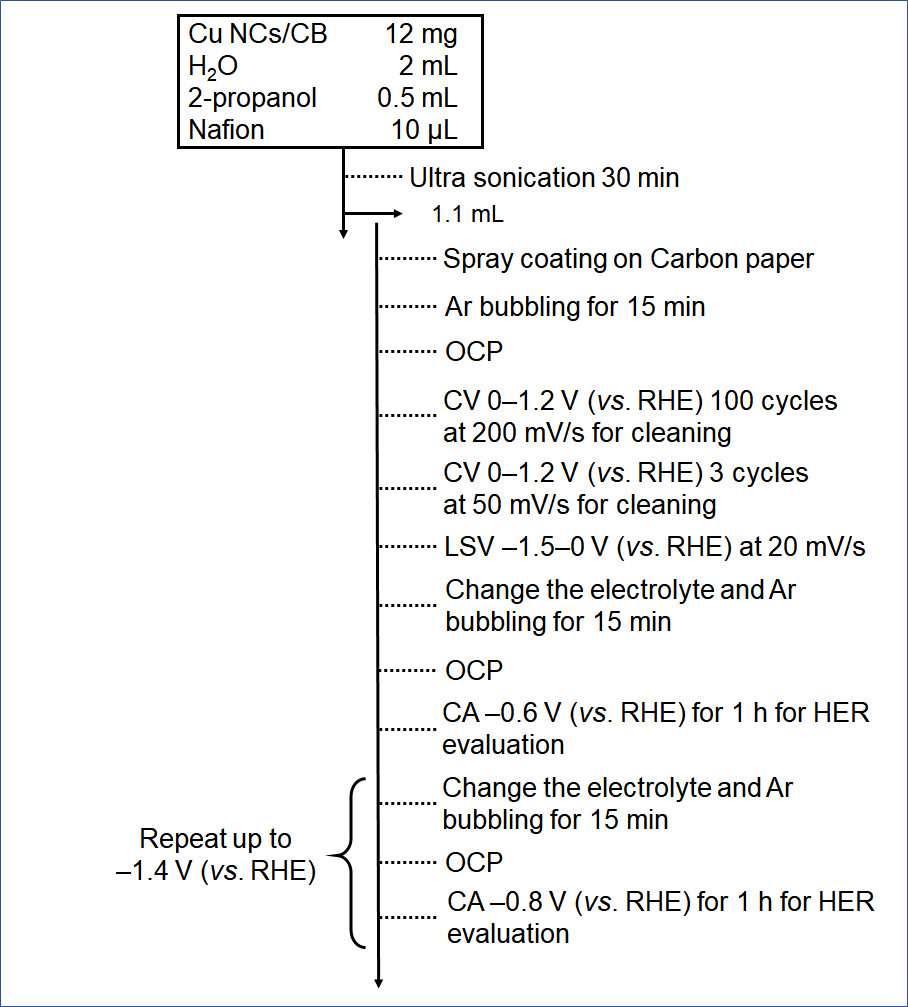
DMSO as internal standard.

Scheme S3. Preparation method of working electrode and electrochemical measurement protocol for HER

X-ray crystallography details

A single crystal was immersed in the cryoprotectant Parabar 10312 (Hampton Research, 34 Journey, Aliso Viejo, CA 92656-3317 USA) and kept at 90 K during diffraction data collection. A Bruker D8 QUEST diffractometer was used to collect the diffraction data for the single crystal using monochromated Mo Kα radiation (λ = 0.71073 Å). Although many crystals from different batches were checked for the diffraction experiment, all of them lacked higher angle data. However, the collected diffraction data was good enough to obtain a structure containing Cu(I) ions, S, P, and a few C atoms, which was solved by SHELXT^S9^ using the intrinsic phasing method in Apex3 Bruker Software Suite.^S10^ Later, during refinement the full crystal structure was completed using the full-matrix least squares method against F2 by SHELXL-2018/3 in Olex2 GUI.^S11^ All the atoms including thiolates, triphenylphosphine and chloroform solvent were refined anisotropically. A few disordered phenyl rings were fixed by AFIX 66. We also assigned the hydrides (H-) based on the q peak, and again confirmed by ESI-MS. There are some alerts in the cif file and we have included our responses here.

For Cu_11_ABT

---------

_vrf_PLAT910_ALERT_3_B

;

PROBLEM: Missing # of FCF Reflection(s) Below Theta (Min).

RESPONSE: Some of the FCF reflections were below theta minimum due to obstruction from the beam stop because of long distance of lattice constant for Cu NCs.

;

_vrf_PLAT416_ALERT_2_B

;

PROBLEM: Short Intra D-H..H-D H..H02N. 1.75 Ang. x,y,z = 1_555 Check.

RESPONSE: Not uncommon for Cu NCs.

;

---------

For Cu_11_PTT,
---------
_vrf_PLAT987_ALERT_1_B
;
PROBLEM: The Flack x is >> 0 - Do a BASF/TWIN Refinement.
RESPONSE: The Flack parameter is near to zero [0.064(3)]. And it is not important to determine absolute configuration.
;
---------

Statistical analysis

We included error bars in the Faradic efficiency (FE) plots, with each error bar representing the standard deviation (SD) from the mean of three experimental measurements (n=3).

Table S1. Crystal data and structure refinement parameters of Cu_11_PTT NC.

| Identification code | Cu11PTT |
| --- | --- |
| Empirical formula | C_170.9_H_152.94_Cu_11_P_6_S_8.98_ |
| CCDC number | 2410866 |
| Formula weight | 3368.46 |
| Temperature/K | 90.15 |
| Crystal system | hexagonal |
| Space group | *P*6_3_ |
| a/Å | 33.4055(6) |
| b/Å | 33.4055(6) |
| c/Å | 27.3177(8) |
| α/° | 90 |
| β/° | 90 |
| γ/° | 120 |
| Volume/Å^3^ | 26400.4(12) |
| Z | 6 |
| ρ_calc_/g cm^-3^ | 1.275 |
| μ/mm^-1^ | 1.507 |
| F(000) | 10387.0 |
| Crystal size/mm^3^ | 0.799 × 0.663 × 0.433 |
| Radiation | MoKα (λ = 0.71073) |
| 2Θ range for data collection/° | 3.724to 52.742 |
| Index ranges | -39 ≤ h ≤ 41, -41 ≤ k ≤ 40, -34 ≤ l ≤ 31 |
| Reflections collected | 316434 |
| Independent reflections | 35369 [R_int_ = 0.0545, R_sigma_ = 0.0320] |
| Data/restraints/parameters | 35369/208/1536 |
| Goodness-of-fit on F^2^ | 1.015 |
| Final R indexes [I>=2σ (I)] | R_1_ = 0.0333, wR_2_ = 0.0776 |
| Final R indexes [all data] | R_1_ = 0.0389, wR_2_ = 0.0807 |
| Largest diff. peak/hole / e Å^-3^ | 0.61/-0.40 |

Table S2. Crystal data and structure refinement parameters of Cu_11_ABT NC.

| Identification code | Cu11ABT |
| --- | --- |
| Empirical formula | C_162_H_144_Cu_11_N_9_P_6_S_9_ |
| CCDC number | 2410867 |
| Formula weight | 3390.15 |
| Temperature/K | 90.15 |
| Crystal system | triclinic |
| Space group | P-1 |
| a/Å | 19.8301(5) |
| b/Å | 21.2225(6) |
| c/Å | 24.2471(7) |
| α/° | 79.3210(10) |
| β/° | 67.4680(10) |
| γ/° | 89.7810(10) |
| Volume/Å^3^ | 9236.7(4) |
| Z | 2 |
| ρ_calc_/g cm^-3^ | 1.219 |
| μ/mm^-1^ | 1.437 |
| F(000) | 3464.0 |
| Crystal size/mm^3^ | 0.188 × 0.102 × 0.084 |
| Radiation | MoKα (λ = 0.71073) |
| 2Θ range for data collection/° | 3.836 to 51.364 |
| Index ranges | -24 ≤ h ≤ 24, -25 ≤ k ≤ 25, -29 ≤ l ≤ 29 |
| Reflections collected | 108623 |
| Independent reflections | 34783 [R_int_ = 0.1012, R_sigma_ = 0.1188] |
| Data/restraints/parameters | 34783/246/1459 |
| Goodness-of-fit on F^2^ | 1.000 |
| Final R indexes [I>=2σ (I)] | R_1_ = 0.0604, wR_2_ = 0.1263 |
| Final R indexes [all data] | R_1_ = 0.1137, wR_2_ = 0.1459 |
| Largest diff. peak/hole / e Å^-3^ | 0.67/-0.68 |

Table S3. Crystal data and structure refinement parameters of Cu_18_PTT NC.

| Identification code | Cu18PTT |
| --- | --- |
| Empirical formula | C_212.99_H_196.99_Cu_17.98_P_6.02_S_14.98_ |
| CCDC number | 2410868 |
| Formula weight | 4567.4888 |
| Temperature/K | 90.15 |
| Crystal system | triclinic |
| Space group | P-1 |
| a/Å | 22.2759(6) |
| b/Å | 25.3548(7) |
| c/Å | 27.6054(9) |
| α/° | 62.8690(10) |
| β/° | 72.9100(10) |
| γ/° | 72.8270(10) |
| Volume/Å^3^ | 13022.8(7) |
| Z | 2 |
| ρ_calc_/g cm^-3^ | 1.164 |
| μ/mm^-1^ | 1.630 |
| F(000) | 4653.0 |
| Crystal size/mm^3^ | 0.707 × 0.354 × 0.284 |
| Radiation | MoKα (λ = 0.71073) |
| 2Θ range for data collection/° | 4.202 to 62.614 |
| Index ranges | -32 ≤ h ≤ 27, -36 ≤ k ≤ 35, -39 ≤ l ≤ 36 |
| Reflections collected | 172188 |
| Independent reflections | 63874 [R_int_ = 0.0371, R_sigma_ = 0.0467] |
| Data/restraints/parameters | 63874/561/2054 |
| Goodness-of-fit on F^2^ | 1.042 |
| Final R indexes [I>=2σ (I)] | R_1_ = 0.0418, wR_2_ = 0.1154 |
| Final R indexes [all data] | R_1_ = 0.0536, wR_2_ = 0.1215 |
| Largest diff. peak/hole / e Å^-3^ | 1.02/-0.72 |


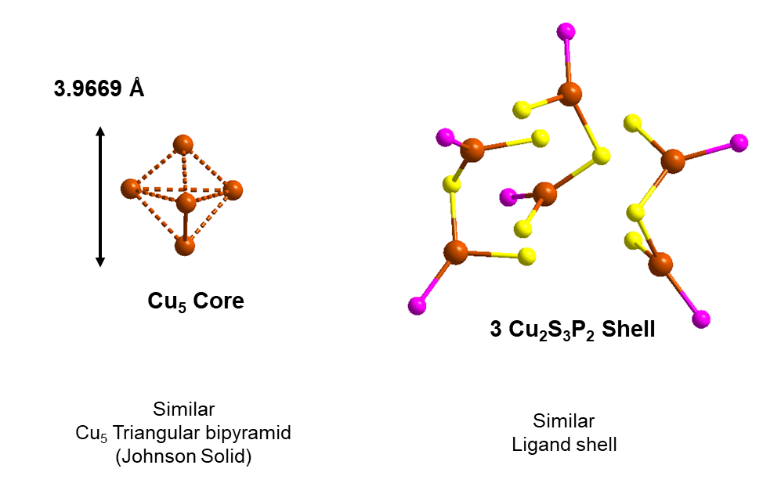


**Figure S1.** Structural architecture of the reported Cu_11_TBBT NC. Hydrogen atoms and part of the ligands are removed for clarity. Color legend: Cu, brown; S, yellow; P, violet.


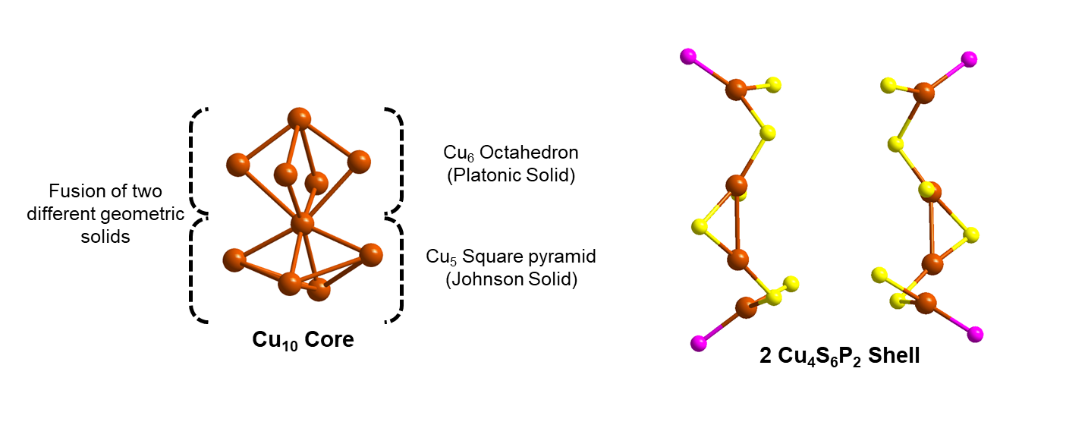


**Figure S2.** Structural architecture of the reported Cu_18_SAdm NC. Hydrogen atoms and part of the ligands are removed for clarity. Color legend: Cu, brown; S, yellow; P, violet.


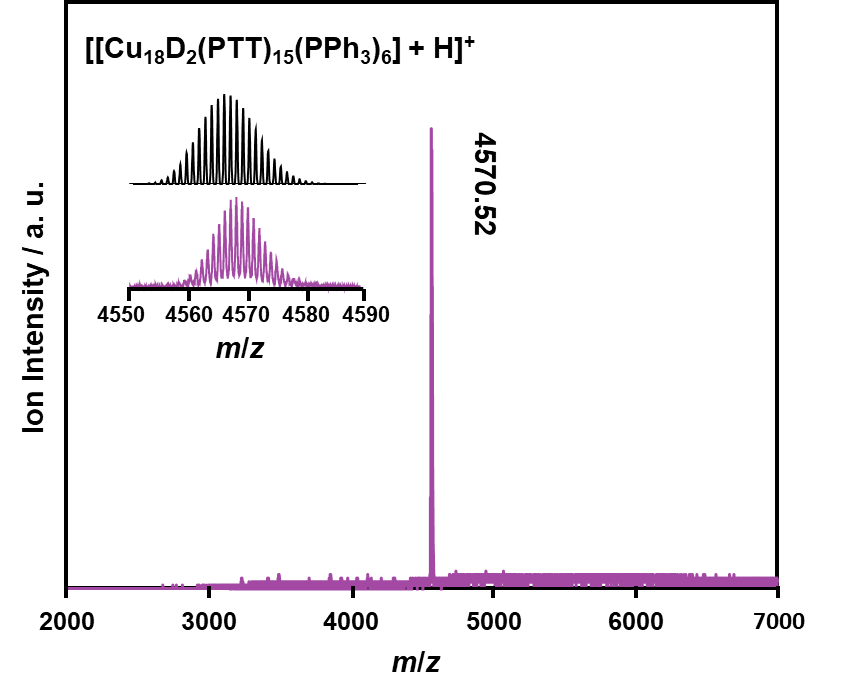


**Figure S3.** Positive mode ESI-MS spectra of [Cu_18_D_2_(PTT)_15_(PPh_3_)_6_] NC.


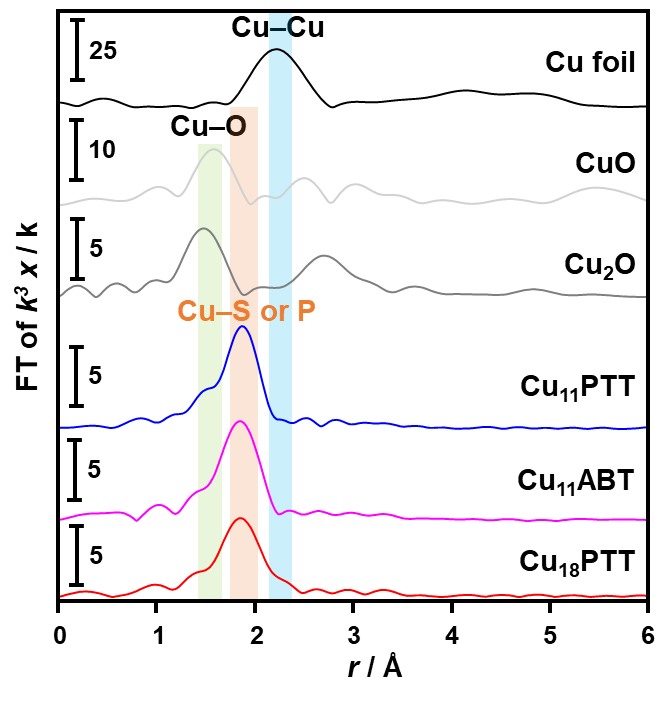


**Figure S4.** Cu-K edge FT-EXAFS data of all Cu NCs.


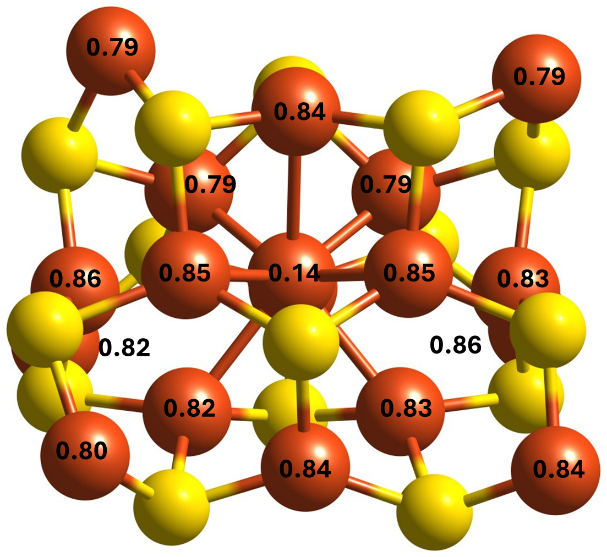


**Figure S5.** Badar charge analysis of individual Cu atoms present in Cu_18_PTT NC. Color legend: Cu, brown; S, yellow.

**
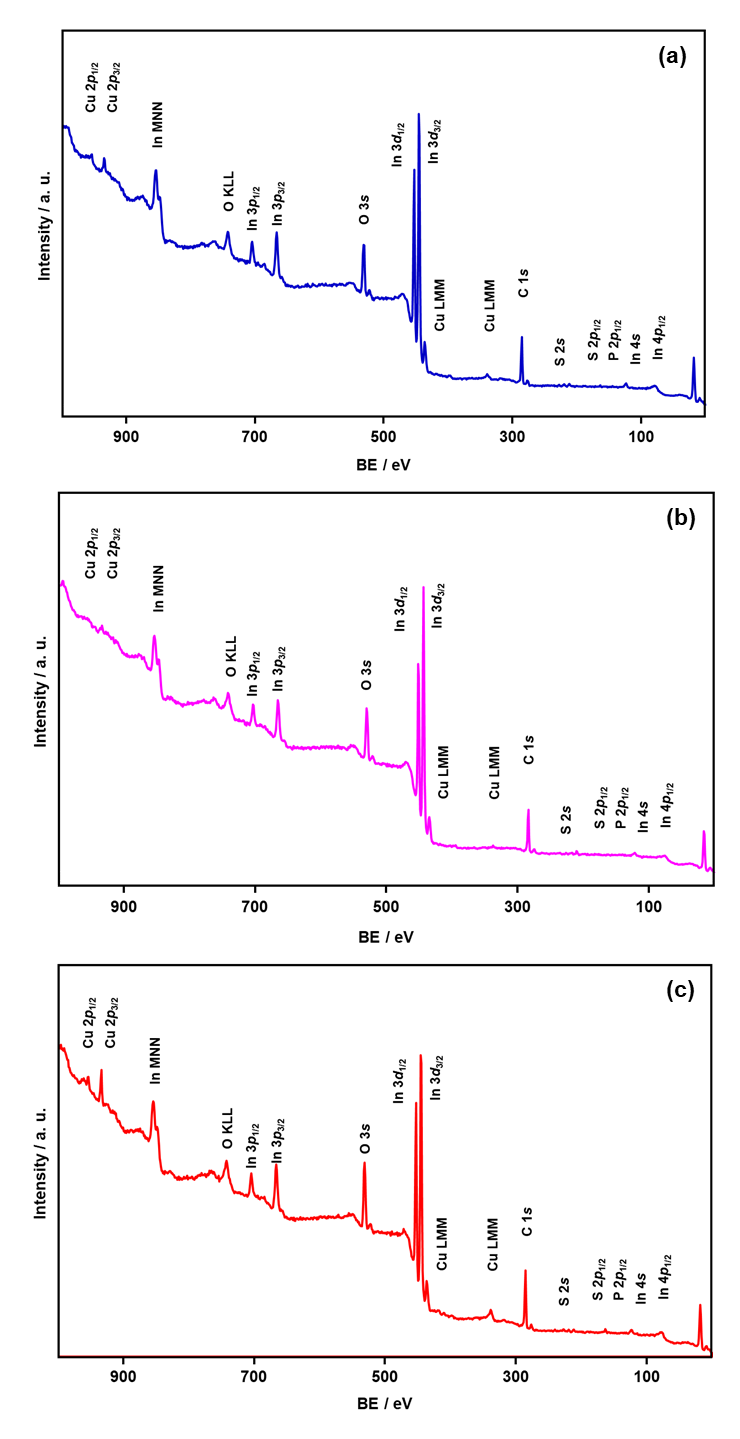
**

**Figure S6.** XPS survey spectrum of (a) Cu_11_PTT, (b) Cu_11_ABT and (c) Cu_18_PTT NCs.


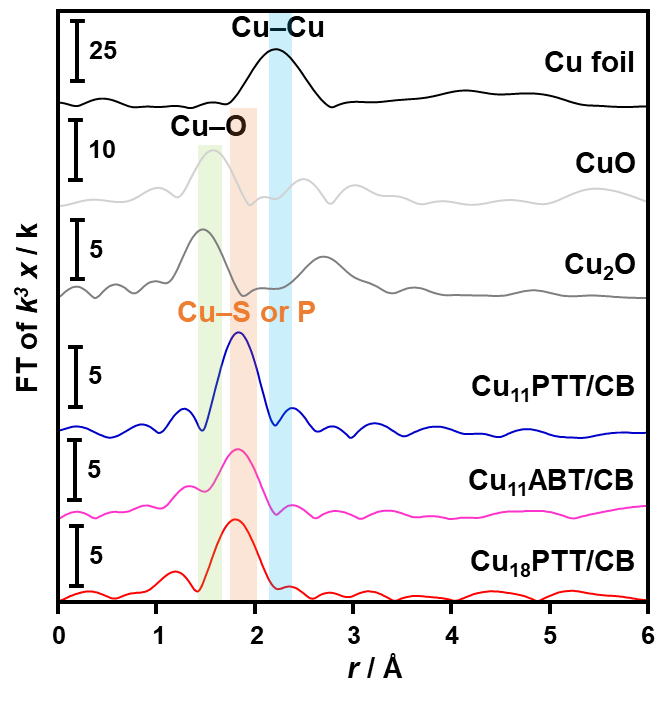


**Figure S7.** FT-EXAFS data of loaded NCs on CB surface.


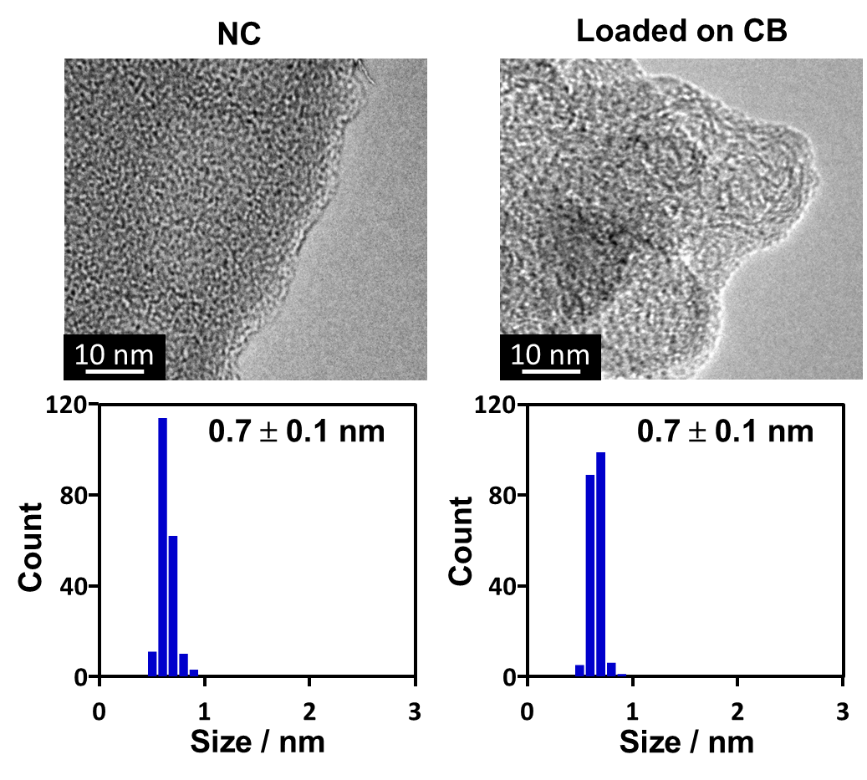


**Figure S8.** TEM images of Cu_11_PTT NCs and Cu_11_PTT loaded on CB.


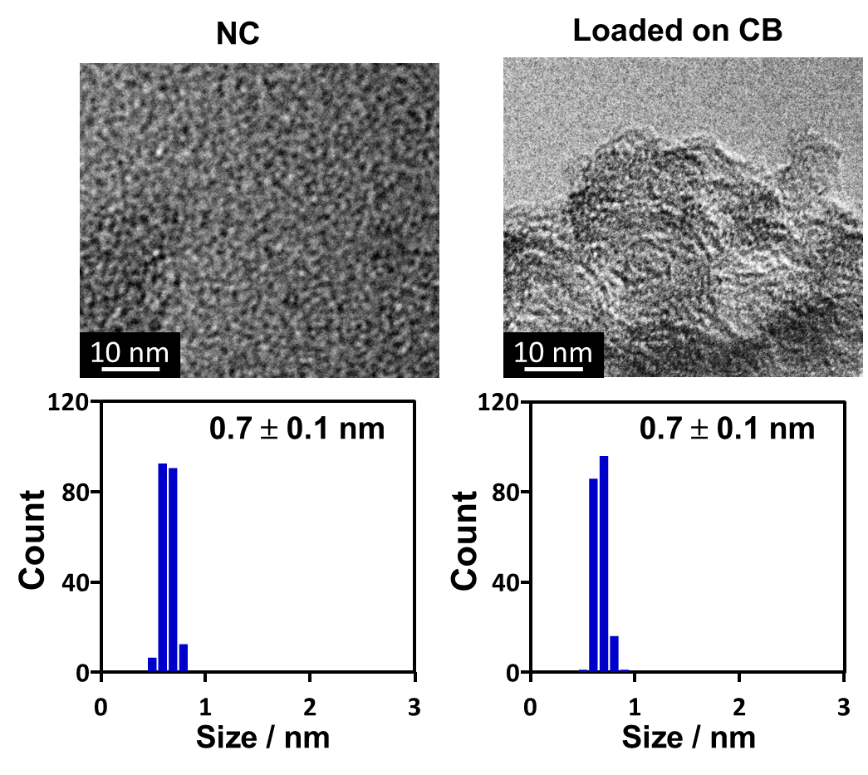


**Figure S9.** TEM images of Cu_11_ABT NCs and Cu_11_ABT loaded on CB.


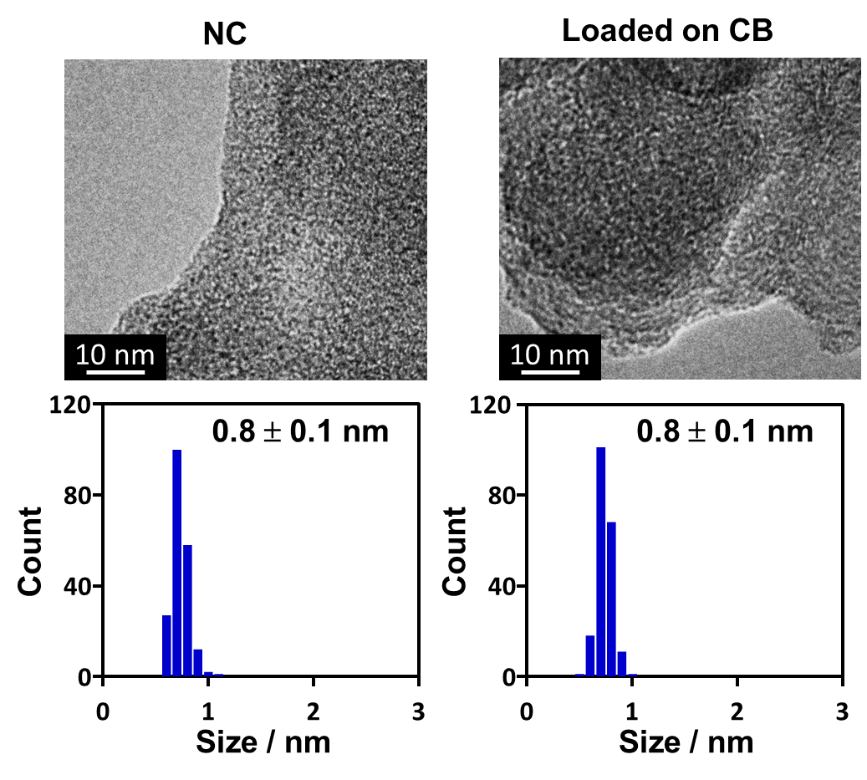


**Figure S10.** TEM images of Cu_18_PTT NCs and Cu_18_PTT loaded on CB.


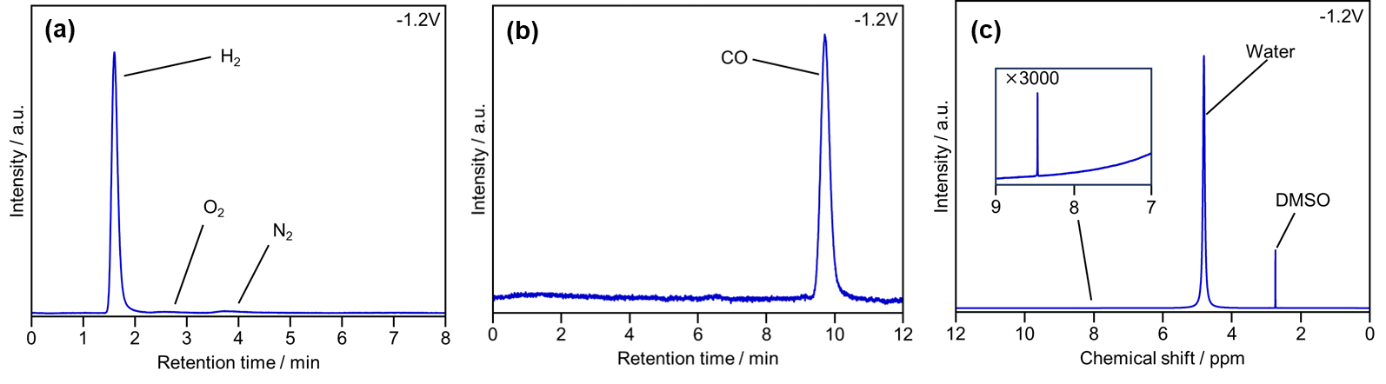


**Figure S11.** The raw data for electrocatalytic CO_2_RR results. (a) GC-TCD, (b) GC-FID and (c) ^1^H NMR spectra after applied potential at −1.2 V vs. RHE for 1 h in 0.1 M KHCO_3_ aq. under CO_2_ flow on Cu_11_PTT NC loaded catalysts.


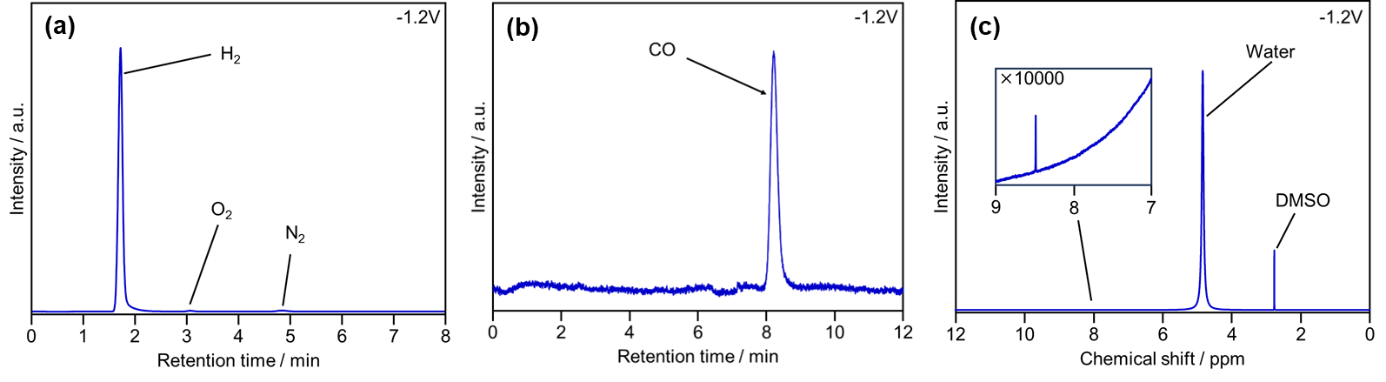


**Figure S12.** The raw data for electrocatalytic CO_2_RR results. (a) GC-TCD, (b) GC-FID and (c) ^1^H NMR spectra after applied potential at −1.2 V vs. RHE for 1 h in 0.1 M KHCO_3_ aq. under CO_2_ flow on Cu_11_ABT NC loaded catalysts.


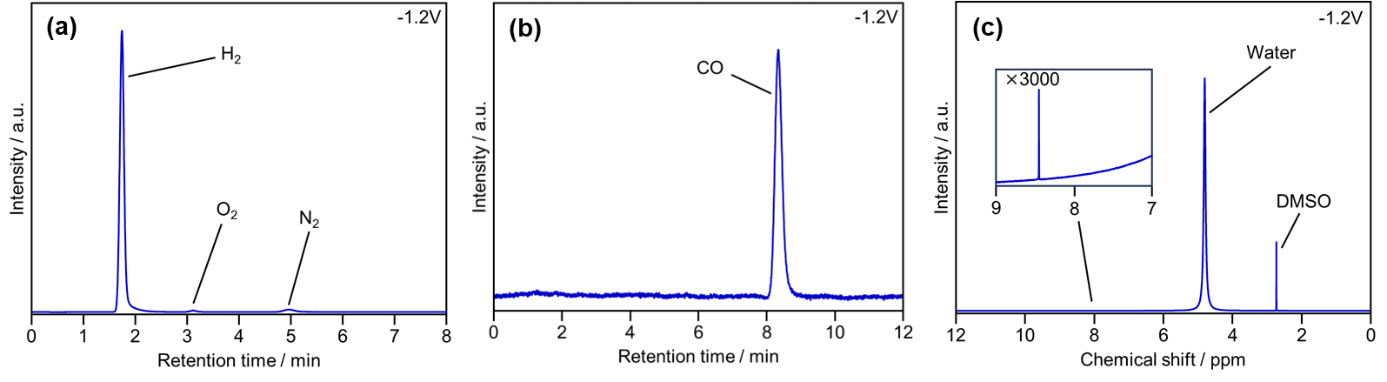


**Figure S13.** The raw data for electrocatalytic CO_2_RR results. (a) GC-TCD, (b) GC-FID and (c) ^1^H NMR spectra after applied potential at −1.2 V vs. RHE for 1 h in 0.1 M KHCO_3_ aq. under CO_2_ flow on Cu_18_PTT NC loaded catalysts.


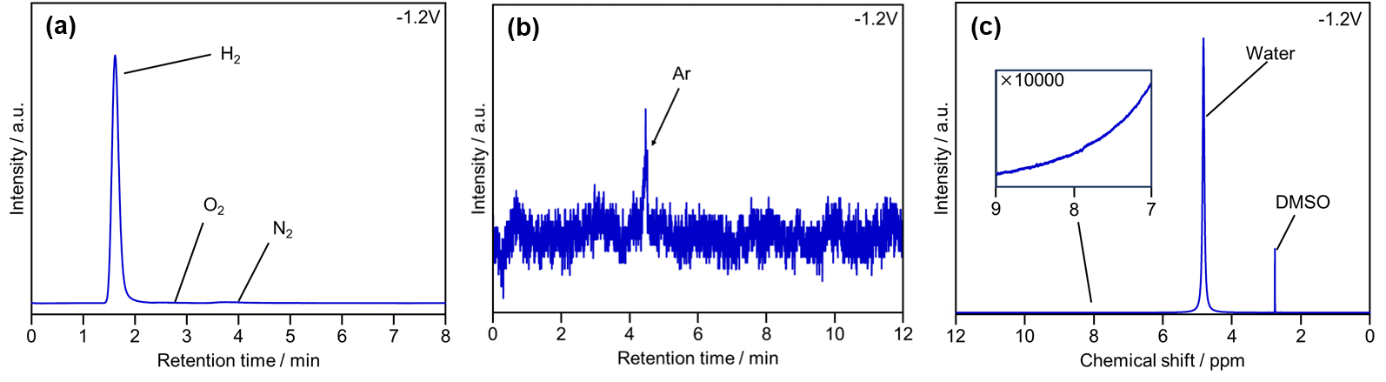


**Figure S14.** The raw data for electrocatalytic CO_2_RR results. (a) GC-TCD, (b) GC-FID and (c) ^1^H NMR spectra after applied potential at −1.2 V vs. RHE for 1 h in 0.1 M KHCO_3_ aq. under Ar flow on Cu_11_PTT NC loaded catalysts.


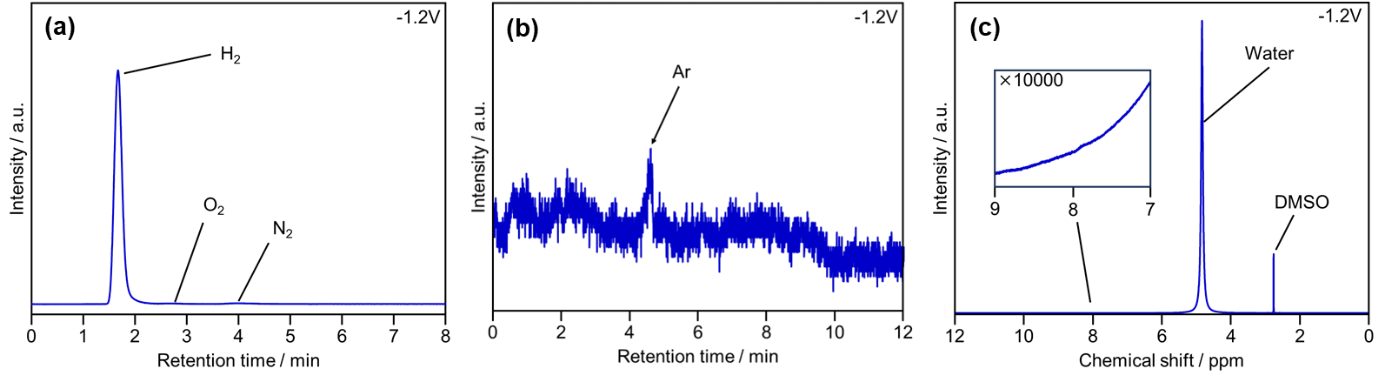


**Figure S15.** The raw data for electrocatalytic CO_2_RR results. (a) GC-TCD, (b) GC-FID and (c) ^1^H NMR spectra after applied potential at −1.2 V vs. RHE for 1 h in 0.1 M KHCO_3_ aq. under Ar flow on Cu_11_ABT NC loaded catalysts.


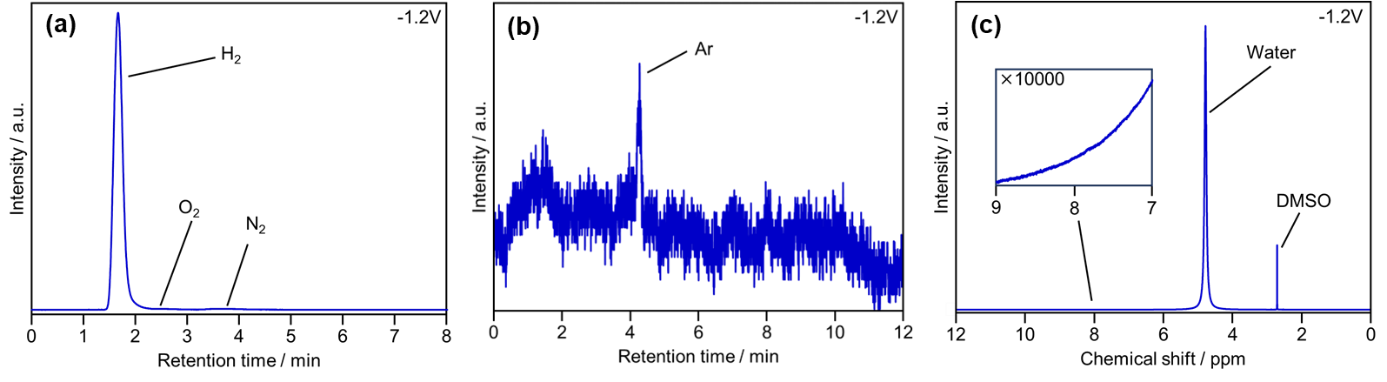


**Figure S16.** The raw data for electrocatalytic CO_2_RR results. (a) GC-TCD, (b) GC-FID and (c) ^1^H NMR spectra after applied potential at −1.2 V vs. RHE for 1 h in 0.1 M KHCO_3_ aq. under Ar flow on Cu_18_PTT NC loaded catalysts.


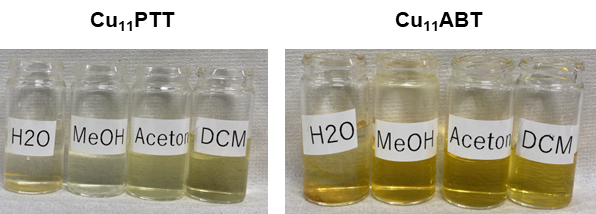


**Figure S17.** Solubility changes of these NCs due to their different functional groups present in the outer surface which influence the hydrophilicity.


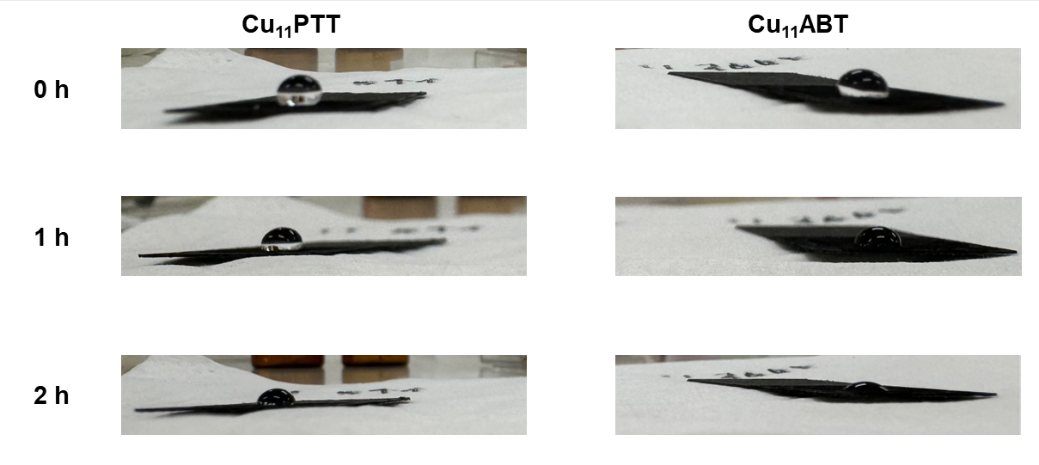


**Figure S18.** Drop-test on carbon paper loaded specific NCs with water droplet.


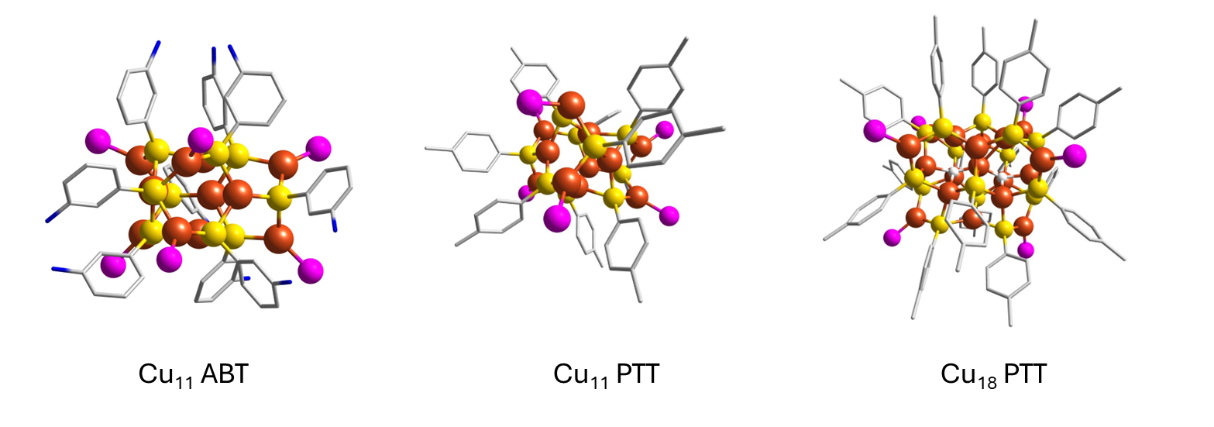


**Figure S19.** Theoretically optimized structures. Hydrogen atoms are removed from the ligands for clarity. Color legend: Cu, brown; S, yellow; P, violet; and C, grey stick.


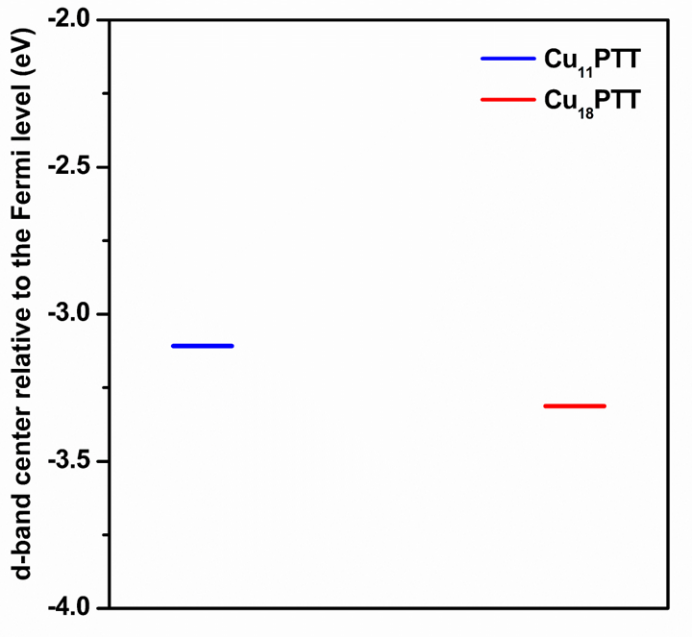


**Figure S20.** Change in the d-band center relative to the Fermi level for Cu_11_PTT and Cu_18_PTT NCs.


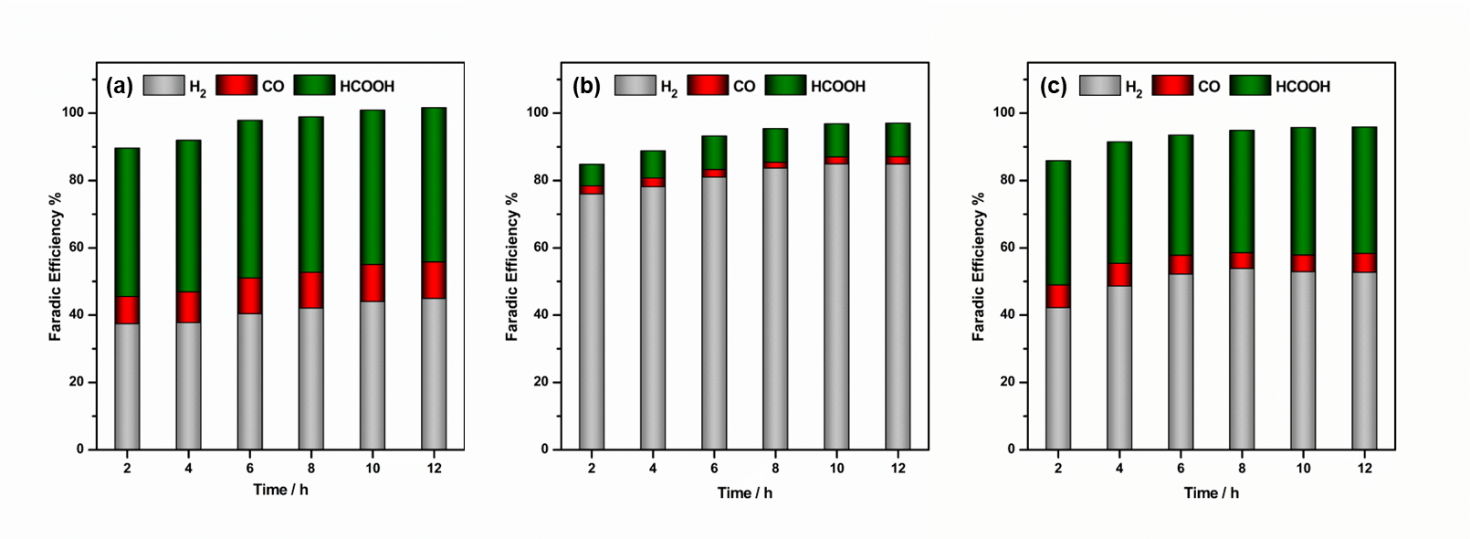


**Figure S21.** Long term chronoamperometric measurements of (a) Cu_11_PTT, (b) Cu_11_ABT and (c) Cu_18_PTT NCs loaded catalysts at -1.2 V (vs. RHE).


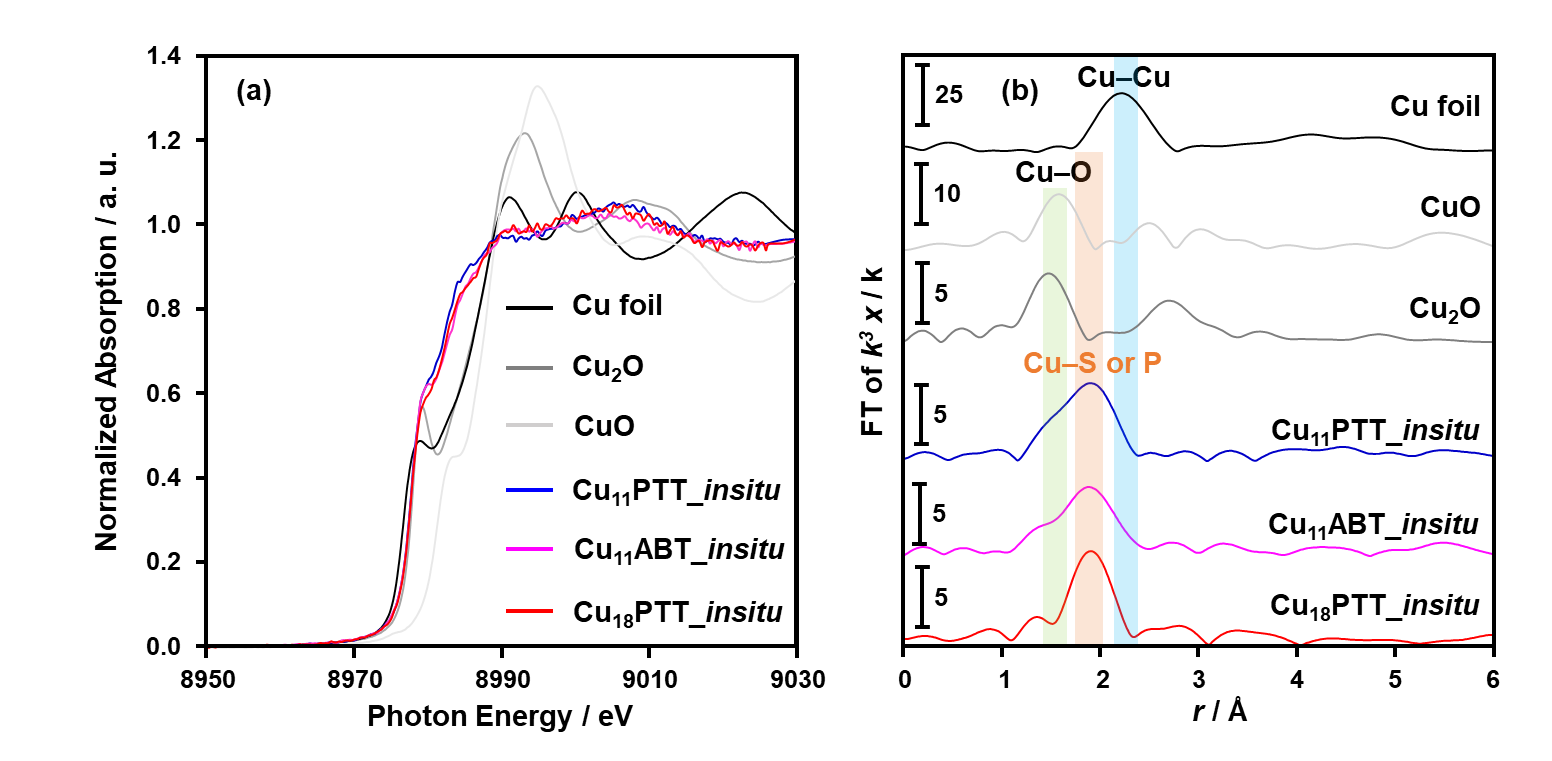


**Figure S22.** (a) Cu-K edge *insitu* XANES spectra and (b) *insitu* FT-EXAFS data of all catalysts. The peak shapes of i*nsitu* XANES spectra at ~8995 eV show similar to those of the as-synthesized NCs, indicating almost complete maintenance of their electronic/structures.


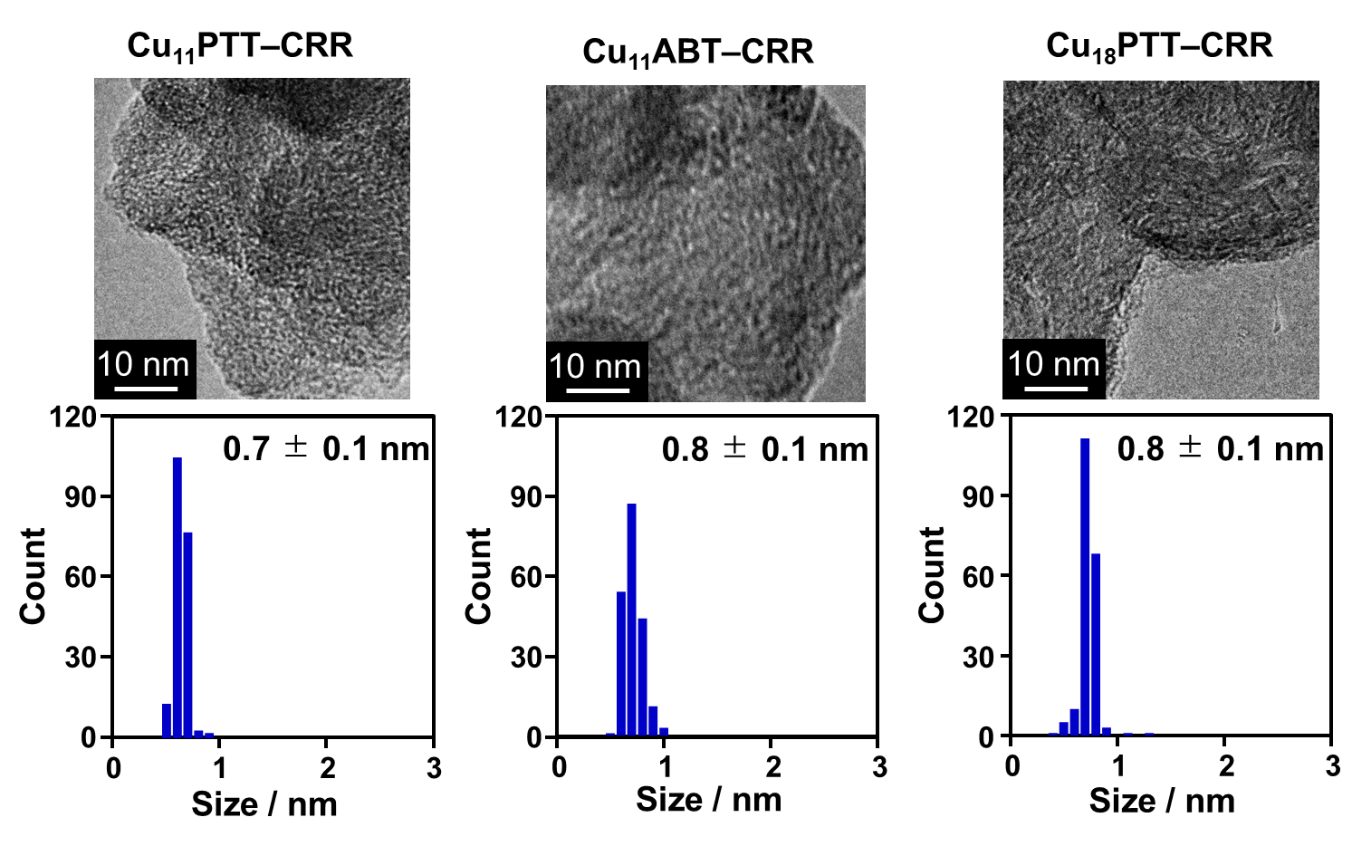


**Figure S23.** TEM images of the individual Cu NCs catalysts after CO_2_RR.


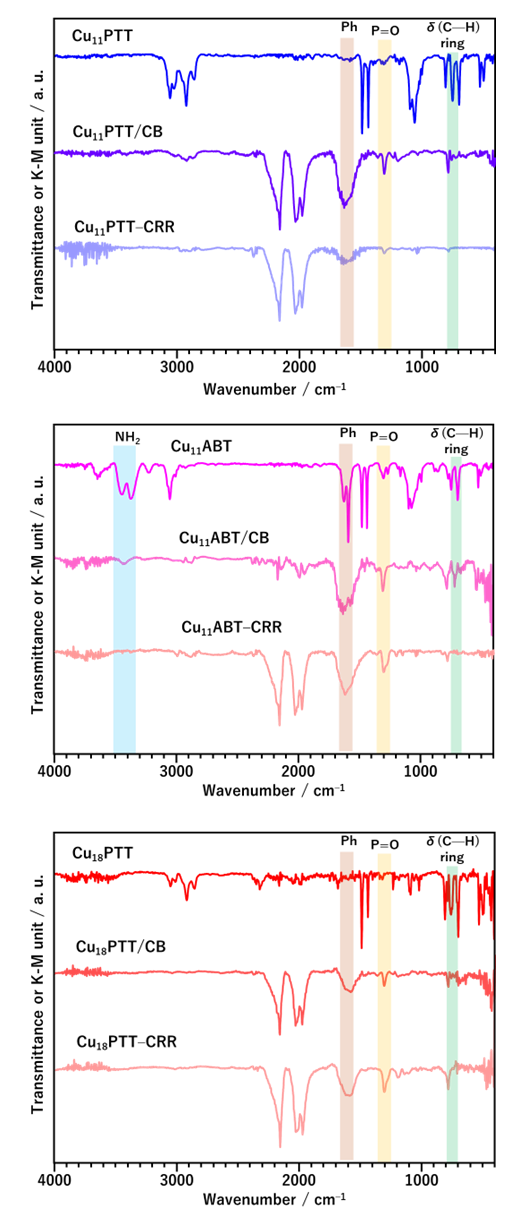


**Figure S24.** FT-IR of individual NCs and catalysts before and after CO_2_RR.

**References**

1. H. Asakura, S. Yamazoe, T. Misumi, A. Fujita, T. Tsukuda, T. Tanaka, *Radiat. Phys. Chem.* **2020**, *175*, 108270.
2. W. Kohn, L. J. Sham, *Phys. Rev.* **1965**, *140*, A1133.
3. G. Kresse, J. Furthmüller, *Phys. Rev.* **1996**, *54*, 11169.
4. P. E. Blöchl, *Phys. Rev.* **1994**, *50*, 17953.
5. J. P. Perdew, K. Burke, M. Ernzerhof, *Phys. Rev. Lett.* **1996**, *77*, 3865.
6. G. Kresse, J. Furthmüller, J. Hafner, *Phys. Rev.* **1994**, *50*, 13181.
7. S. Grimme, J. Antony, S. Ehrlich, H. Krieg, *J. Chem. Phys.* **2010**, 132.
8. J. K. Nørskov, J. Rossmeisl, A. Logadottir, L. Lindqvist, J. R. Kitchin, T. Bligaard, H. Jonsson, *J. Phys. Chem.* **2004**, *108*, 17886-17892.
9. G. M. Sheldrick, *Acta Crystallogr., Sect. C: Struct. Chem.* **2015**, *71*, 3-8.
10. Bruker APEX3, v2019.1–0, Bruker AXS Inc., Madison, WI, USA, (2019).
11. O. V. Dolomanov, L. J. Bourhis, R. J. Gildea, J. A. Howard, H. Puschmann, *J. Appl. Crystallogr.* **2009**, *42*, 339-341.
